# Animal models of chemotherapy-induced peripheral neuropathy: A machine-assisted systematic review and meta-analysis

**DOI:** 10.1371/journal.pbio.3000243

**Published:** 2019-05-20

**Authors:** Gillian L. Currie, Helena N. Angel-Scott, Lesley Colvin, Fala Cramond, Kaitlyn Hair, Laila Khandoker, Jing Liao, Malcolm Macleod, Sarah K. McCann, Rosie Morland, Nicki Sherratt, Robert Stewart, Ezgi Tanriver-Ayder, James Thomas, Qianying Wang, Rachel Wodarski, Ran Xiong, Andrew S. C. Rice, Emily S. Sena

**Affiliations:** 1 Centre for Clinical Brain Sciences, University of Edinburgh, Edinburgh, United Kingdom; 2 Pain Research, Department of Surgery and Cancer, Imperial College London, London, United Kingdom; 3 Department of Anaesthesia, Critical Care & Pain, University of Edinburgh, Edinburgh, United Kingdom; 4 Division of Population Health and Genomics, University of Dundee, Dundee, United Kingdom; 5 EPPI-Centre, University College London, London, United Kingdom; University of Ottawa, The Ottawa Hospital Research Institute, CANADA

## Abstract

We report a systematic review and meta-analysis of research using animal models of chemotherapy-induced peripheral neuropathy (CIPN). We systematically searched 5 online databases in September 2012 and updated the search in November 2015 using machine learning and text mining to reduce the screening for inclusion workload and improve accuracy. For each comparison, we calculated a standardised mean difference (SMD) effect size, and then combined effects in a random-effects meta-analysis. We assessed the impact of study design factors and reporting of measures to reduce risks of bias. We present power analyses for the most frequently reported behavioural tests; 337 publications were included. Most studies (84%) used male animals only. The most frequently reported outcome measure was evoked limb withdrawal in response to mechanical monofilaments. There was modest reporting of measures to reduce risks of bias. The number of animals required to obtain 80% power with a significance level of 0.05 varied substantially across behavioural tests. In this comprehensive summary of the use of animal models of CIPN, we have identified areas in which the value of preclinical CIPN studies might be increased. Using both sexes of animals in the modelling of CIPN, ensuring that outcome measures align with those most relevant in the clinic, and the animal’s pain contextualised ethology will likely improve external validity. Measures to reduce risk of bias should be employed to increase the internal validity of studies. Different outcome measures have different statistical power, and this can refine our approaches in the modelling of CIPN.

## Abstract 2

This systematic review assesses limitations in the experimental design of studies modelling peripheral neuropathy induced by chemotherapy. The data allow the authors to provide guidance on experimental design to improve the robustness and validity of future studies.

## Author summary

Many frequently used and effective cancer chemotherapies can cause a disabling side effect that features pain, numbness, tingling, and sensitivity to cold and heat in the extremities known as chemotherapy-induced peripheral neuropathy (CIPN). There are currently no effective therapies to treat or prevent this condition, and animal models have been developed to address this. It is important that experiments using animal models of CIPN are robust and valid if they are to effectively help patients. We used a systematic approach to identify all 337 studies that have been published describing the use of animal models of CIPN. We were able to identify that many studies are imperfect in their experimental design, use only male animals, and assess outcomes with limited relevance to the human condition. Based on a meta-analysis, we provide guidance to the CIPN animal modelling community to guide future experiments that may improve their utility and validity.

## Introduction

Chemotherapy-induced peripheral neuropathy (CIPN) is a disabling side effect of many frequently used and effective cancer chemotherapeutic agents and is known to impair daily function and diminish quality of life [1]. Frequently used chemotherapeutic agents reported to cause neurotoxic effects include platinum derivatives, taxanes [2], vinca alkaloids, epothilones, and also newer agents (e.g., thalidomide and bortezomib) [3]. The predominant sensory phenotype in patients exposed to oxaliplatin or docetaxel is distal symmetrical sensory loss affecting both upper and lower extremities. Symptoms of sensory disturbance reported by patients include paraesthesiae, numbness or tingling, and, less frequently, pain and cold allodynia [4]. CIPN can present clinically in 2 distinct forms: acute and chronic. The acute form is a chemotherapy dose-related, and often dose-limiting, polyneuropathy, which in many cases resolves in patients once the chemotherapy ceases. In some patients, this will persist, with other patients only developing symptoms after treatment has finished. A chronic, often painful, distal sensory neuropathy is still present in 33% of patients 1 year after completion of treatment [5]. No preventive or curative disease modifying treatments exist, and therefore there is a pressing need for more effective treatments [6].

Animal models of CIPN are used to investigate the pathophysiology of CIPN and to test potential therapies [7]. Frequently, chemotherapeutic agents are administered to induce a sensory neuropathy, and behavioural tests are used to assess induced sensory phenomena, such as evoked pain, and locomotor activity. Unfortunately, the conventional paradigm for drug development, in which findings are translated from preclinical animal research to clinical treatments, has been characterised by a lack of success [8,9]. Metaresearch from preclinical stroke research suggests that limitations in experimental design, conduct, analysis, and reporting—such as failure to carry out blinded assessment of outcome, randomisation and allocation concealment—may be impeding the development of effective therapies [10–13]. This led to the development of evidence-based guidelines for scientists [14]. These recommendations have been highly successful in transforming the reporting of measures to reduce risk of bias in the preclinical stroke field [15].

We have used a systematic review, in which we systematically identify and appraise all available evidence relevant to a predefined research question to provide a complete and unbiased summary of available evidence. We seek to establish the extent to which limited experimental biases influence the preclinical CIPN literature and to provide evidence to inform tactics to increase the scientific validity of this research. Our aim is to provide a systematic overview of research in the field of in vivo animal modelling of CIPN, with a focus on the reporting of pain-related behavioural outcome measures, to provide useful information for preclinical researchers wishing to improve the design of experiments and refine the in vivo modelling of painful neuropathy.

## Results

### Identification of publications

**Fig 1:**
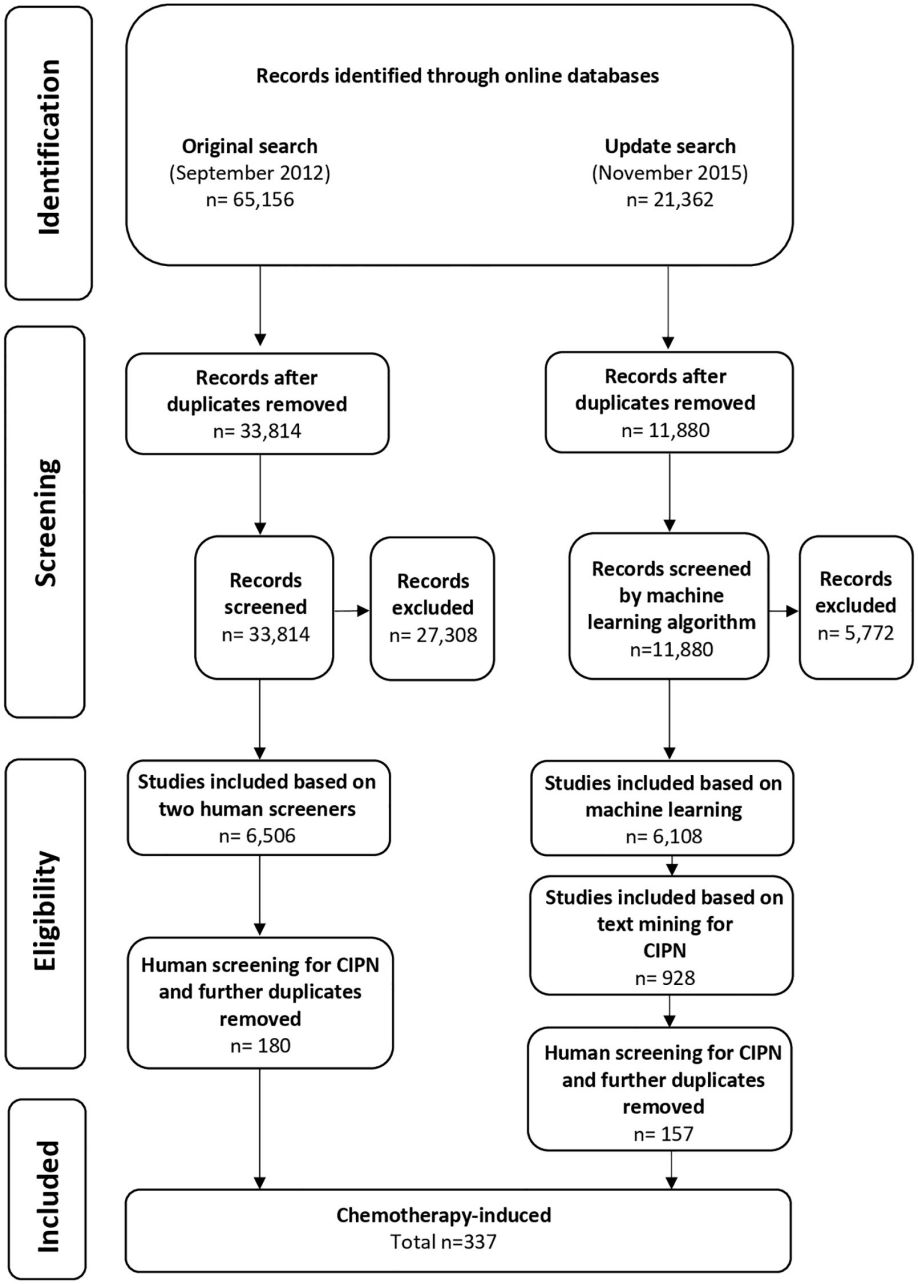
Flow diagram of included studies. CIPN, chemotherapy-induced peripheral neuropathy.

Our initial systematic search (September 2012) identified 33,184 unique publications, of which 6,506 were identified as reporting in vivo models of painful neuropathy. This screening stage took 18 person months; 180 of these publications reported models of CIPN (Fig 1).

In the updated search (November 2015), we identified a further 11,880 publications. Using machine learning and text mining, we identified 6,108 publications as likely to report models of neuropathic pain, and 928 of these reported models of CIPN. In a random 10% sample of screened publications (*n* = 1,188), the classifier with the best fit—using stochastic gradient descent—had a screening performance of 97% sensitivity, 67% specificity, and 56% precision. Further details of the different machine-learning approaches applied are available [16]. Of the 928 studies identified to report animal models of CIPN, 157 met our inclusion criteria.

From both searches, a total of 337 unique publications are included in this review. The rate of new publications per year is shown in S1 Fig. Metadata from the 337 publications included in this study are available on the Open Science Framework (OSF; https://doi.org/10.17605/OSF.IO/ZJEHY). To address concerns that the systematic search is dated we performed a cumulative meta-analysis, in a post hoc analysis, of the effect sizes and tau^2^ estimates (an estimate of between-study heterogeneity), ordered by year of publication. It appears that the data are mature and stable from around 250 studies onwards (S2 Fig).

To investigate sources of heterogeneity we divided the reporting of results by type of study (i.e., modelling experiments or intervention experiments), and by type of outcome measures reported (i.e., pain-related behaviours or other behaviours). Therefore, we have 4 datasets: (i) Data set 1a—modelling of CIPN and reporting pain-related behavioural outcome measures, (ii) Data set 1b—modelling of CIPN and reporting other behavioural outcome measures, (iii) Data set 2a –effects of interventions in animal models of CIPN and reporting pain-related behavioural outcome measures, and (iv) Data set 2b—effects of interventions in animal models of CIPN and reporting other behavioural outcome measures.

### Outcome measures

**Fig 2:**
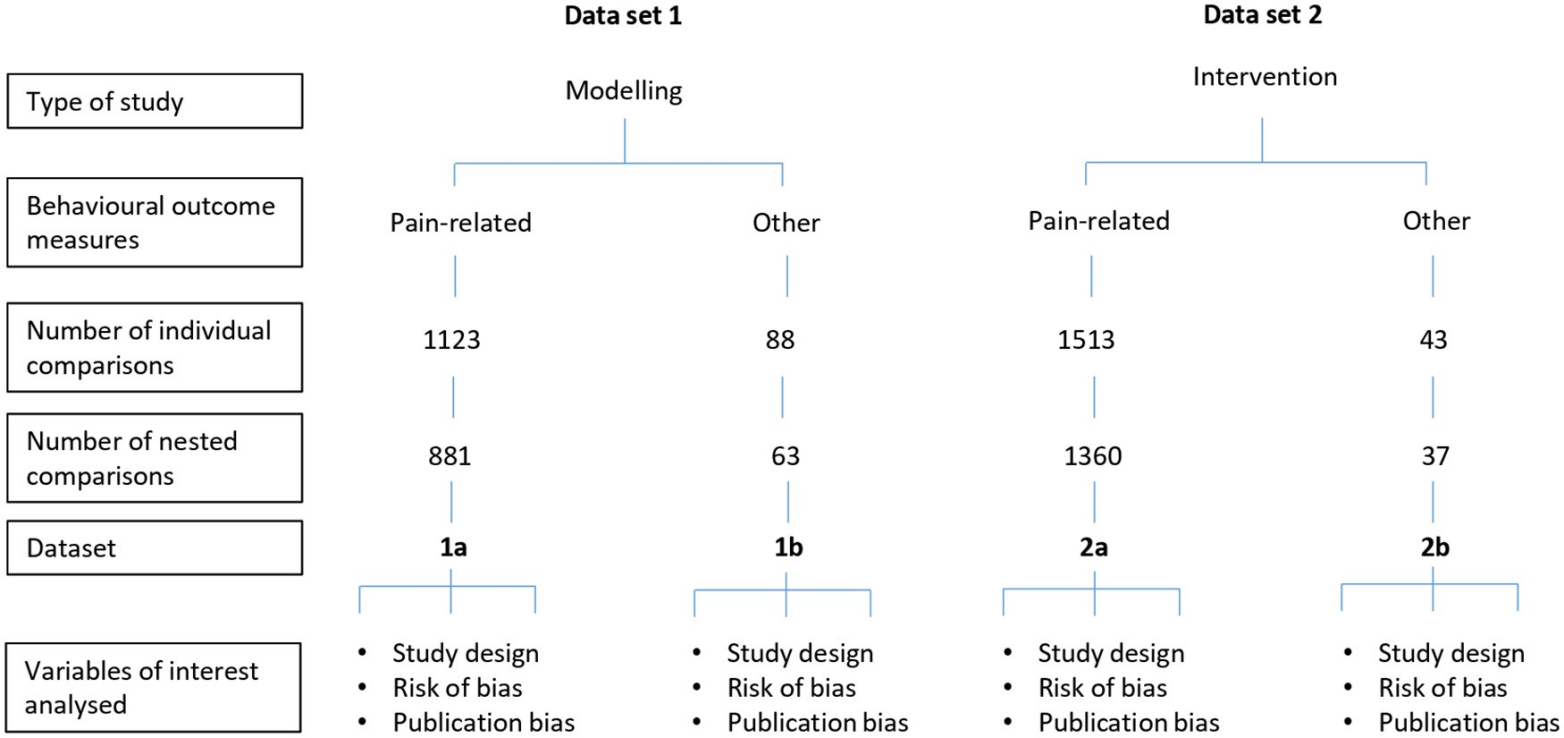
Description and summary of data sets included in this review.

Across the 337 publications included, we extracted all behavioural outcome measure data. Pain-related outcome measures included evoked limb withdrawal to stimuli (mechanical, heat, cold, and/or dynamic mechanical touch), evoked limb withdrawal and/or vocalisation to pressure stimuli, evoked tail withdrawal to stimuli (cold, heat, and/or pressure), and complex behaviours, e.g., burrowing activity. Other outcome measures included assessment of locomotor function, memory, reward, and attention. Pain-related and other outcome measures for both modelling and intervention experiments were analysed separately (Fig 2). The full list of behavioural outcome measures and behavioural tests is given in Tables 1 and 2. The most frequently reported pain-related outcome measure was evoked limb withdrawal to mechanical stimuli, most frequently assessed using monofilaments (Table 1). The most frequently reported other behavioural outcome measure was locomotor function, with the rotarod apparatus used in most cases (Table 2).

**Table 1:** Pain-related behavioural outcome measures across intervention and modelling experiments. Numbers indicate the number of individual comparisons.

Pain-related behavioural outcome	Modelling	Intervention
	Number of comparisons	
**ELW: Mechanical**	**534**	**750**
Dynamic plantar aesthesiometer	1	2
Electronic "von Frey"	66	143
Mechanical monofilament	458	593
Normal non-noxious palpation of the tibia	1	0
Pin prick	8	12
Pinch test	2	2
**ELW: Cold**	191	264
Acetone/ethylchloride/menthol	110	140
Cold plate	73	117
Cold probe	1	0
Cold tolerance	0	1
ELW: cold water	6	6
Limb immersion: cold	1	0
**ELW or vocalisation: Pressure**	**149**	240
ELW or vocalisation: pressure	149	240
**ELW: Heat**	135	146
ELW: radiant heat	101	84
Hot plate	34	61
Paw immersion: heat	0	1
**ETW: Cold**	**43**	**51**
Tail immersion: cold	43	51
**ETW: Heat**	**54**	**44**
Tail flick: heat	33	32
Tail immersion: heat	21	12
**Complex behaviour**	**8**	**12**
AITC-evoked nocifensive behaviour	1	2
Burrowing activity	2	0
Capsaicin-evoked nocifensive behaviour	1	0
Conditioned place preference: pain-related	0	2
Orofacial operant assessment: cold	2	4
Thermal place preference: cold	2	4
**ELW: Dynamic mechanical touch**	**5**	**4**
Rough brush	2	2
Soft brush/cotton tip/ball	3	2
**ETW: Pressure**	**2**	**0**
Tail pressure test	2	0

Frequent descriptions for ELW or vocalisations: pressure (with the Randall-Selitto/paw pressure test). Frequent description for ELW: radiant heat (with Hargreave’s).

**Abbreviations**: AITC, TRPA1 agonist allyl isothiocyanate; ELW, evoked limb withdrawal; ETW, evoked tail withdrawal.

**Table 2:** Other behavioural outcome measures across intervention and modelling experiments. Number of individual comparisons.

Other behavioural outcome	Modelling	Intervention
	Number of comparisons	
**Locomotor function**	**73**	**37**
Rotarod: locomotor function	34	23
Grip test: motor strength	14	1
Locomotor activity	10	6
Open field: distance travelled	4	0
Open field: exploratory behaviour	2	2
Open field: loading on hind limbs	2	2
Open field: immobility	2	0
Open field: locomotor activity	2	0
Catwalk: gait alterations	2	0
Balance beam: motor coordination	1	1
Cannabinoid tetrad test	0	1
Cannabinoid tetrad test: motility	0	1
**Memory**	**8**	0
Novel location preference: preference score	4	0
Novel object preference: preference score	4	0
**Reward**	**4**	6
Conditioned place preference: rewarding effects	4	6
**Attention**	**3**	0
Prepulse inhibition: attention	3	0

### Interventions

A total of 306 different interventions were tested (S3 Fig). Most (80%) were only tested in 1 publication, and the most frequently reported interventions were gabapentin, morphine, and pregabalin, which were reported in 26, 22, and 11 publications, respectively.

### Risk of bias

The reporting of measures to reduce risk of bias was ‘moderate’ across included studies (*n* = 337): 51.3% (*n* = 173) reported blinded assessment of outcome, 28.5% (*n* = 96) reported randomisation to group, 17.8% (*n* = 60) reported animal exclusions, 2.1% (*n* = 7) reported the use of a sample size calculation, and 1.5% (*n* = 5) reported allocation concealment.

Across all included studies, 49.6% (*n* = 167) reported a conflict of interest statement, and 96.7% (*n* = 326) reported compliance with animal welfare regulations (Table 3).

**Table 3:** Reporting of measures to reduce the risk of bias and reporting.

	Risk of bias						Reporting	
	Blinded assessment of outcome	Allocation concealment	Random allocation to group	Sample size calculation	Animal exclusions		Conflict of interest statement	Compliance with animal welfare regulations
% Reporting (Number/337)	51.3 (173)	1.5 (5)	28.5 (96)	2.1 (7)	17.8 (60)		49.6 (167)	96.7 (326)

The methods used to implement randomisation and blinding, and the methods and assumptions for sample size calculations, were rarely reported: 6 publications reported that animals were randomly allocated to experimental groups using randomly generated number sequences, and 2 publications reported that this was done by block randomisation (8.3% of those that reported randomisation; 8 out of 96). One publication reported that randomisation was performed by ‘picking animals randomly from a cage’, which we do not consider a valid method of randomisation [17]. Nine publications reported that blinded outcome assessment was achieved by using a different experimenter to perform assessments, and 2 publications reported that a group code was used (6.4% of those that reported blinding; 11 out of 173). One study reported that allocation concealment was achieved using a coded system (20% of those that reported allocation concealment; 1 out of 5). Methods of sample size calculation were reported by 5 publications (71.4% of those that reported a sample size calculation; 5 out of 7): 3 used published or previous results from the group, and 2 had performed a pilot study to inform sample size calculations.

### Modelling experiments

#### Animal studies modelling CIPN: Pain-related behavioural outcome measures (data set 1a)

In modelling experiments using pain-related behavioural outcome measures, administration of a chemotherapeutic agent led to increased pain-related behaviour compared to sham controls (−2.56 standard deviation [SD] [95% CI −2.71 to −2.41], *n* = 881 comparisons). Species did not account for a significant proportion of the heterogeneity, and therefore mouse and rat experiments were analysed together (mice: −2.63 SD [95% CI −2.86 to −2.39], *n* = 337 comparisons; rats: −2.52 SD [95% CI −2.71 to −2.32], *n* = 544 comparisons; Q = 1.16, df = 1, *p* = 0.28).

#### Study design

**Fig 3:**
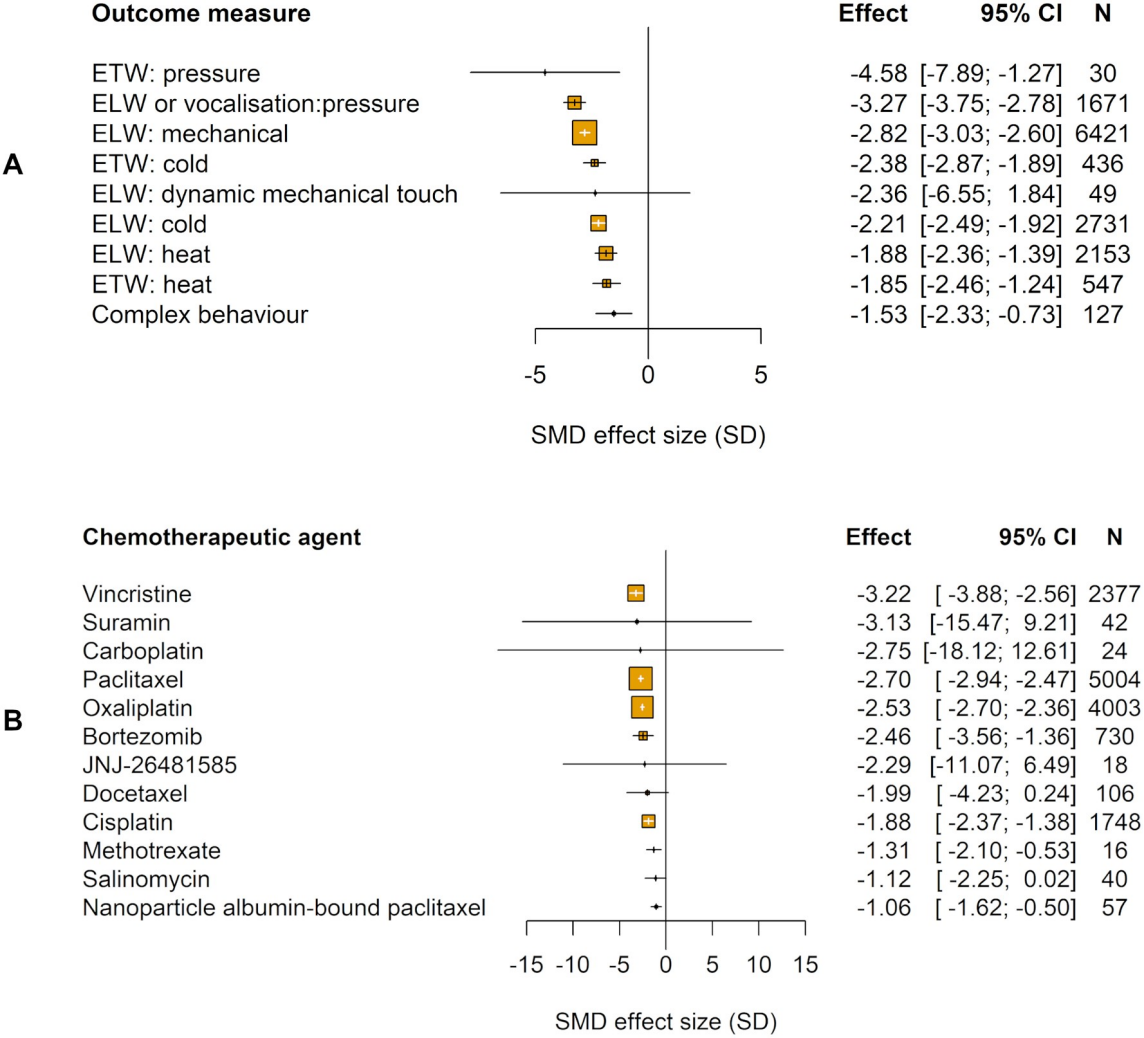
Impact of study design in modelling experiments using pain-related behavioural outcomes (data set 1a). The size of the squares represents the number of nested comparisons that contribute to that data point and the value *N* represents the number of animals that contribute to that data point. (A) Outcome measure accounted for a significant proportion of the heterogeneity. ETW, ELW, and complex behaviours used to measure pain. (B) Chemotherapeutic agent accounted for a significant proportion of the heterogeneity. ELW, evoked limb withdrawal; ETW, evoked tail withdrawal; SMD, standardised mean difference.

The type of pain-related outcome measure accounted for a significant proportion of the heterogeneity (Q = 307.27, df = 8, *p* ≤ 0.01; Fig 3A).

**Fig 4:**
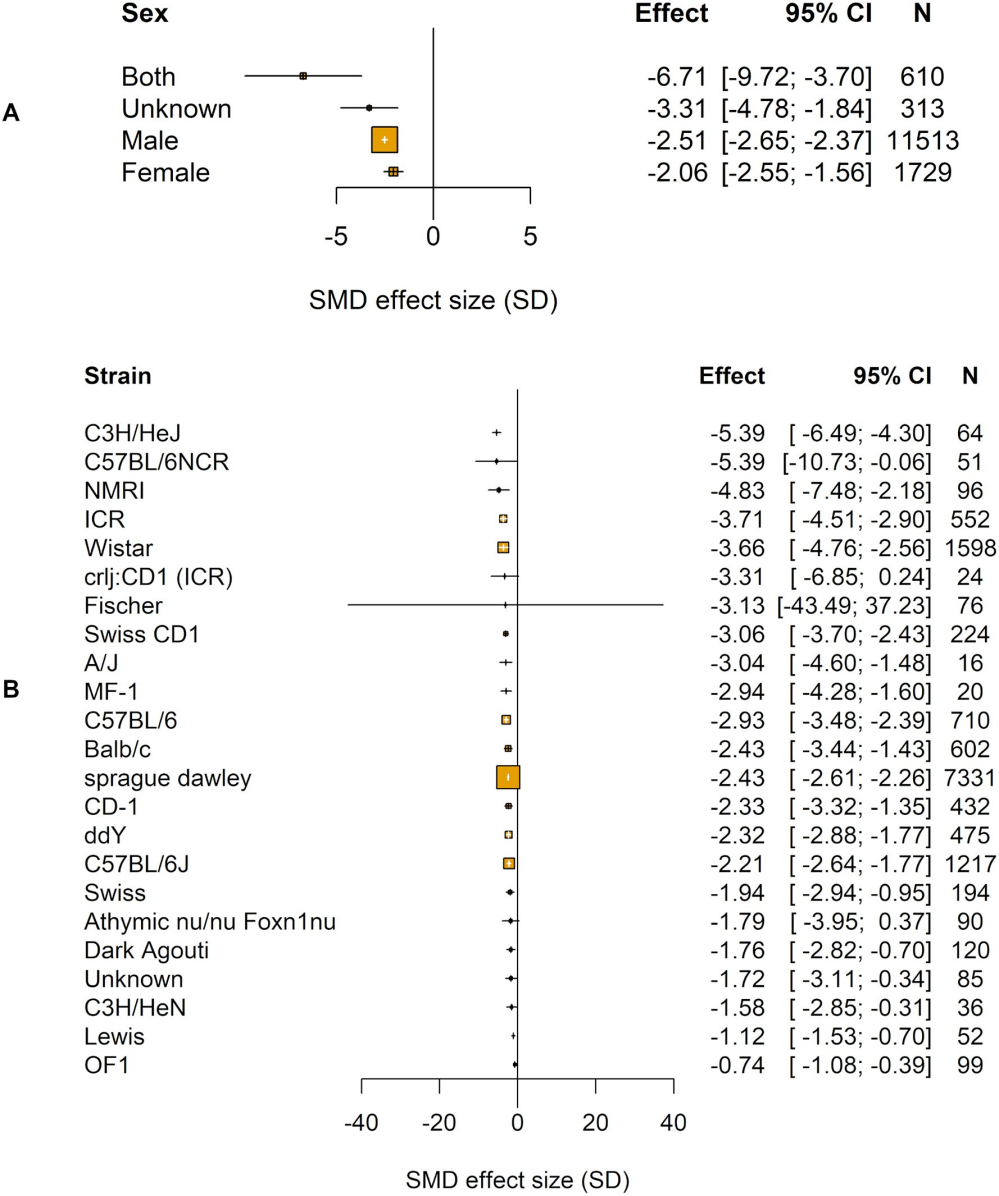
Impact of study design in modelling experiments using pain-related behavioural outcomes (data set 1a). The size of the squares represents the number of nested comparisons that contribute to that data point, and the value *N* represents the number of animals that contribute to that data point. (A) Sex accounted for a significant proportion of the heterogeneity. (B) Strain accounted for a significant proportion of the heterogeneity. SMD, standardised mean difference.

We identified 12 different chemotherapeutic agents used to model CIPN in animals (Table 4). The chemotherapeutic agent used accounted for a significant proportion of the heterogeneity observed (Q = 174.26, df = 11, *p* < 0.01; Fig 3B). Sex accounted for a significant proportion of the heterogeneity (Q = 137.11, df = 3, *p* < 0.01; Fig 4A).

**Table 4:** Model details including chemotherapeutic agents, route of administration, median cumulative dose, and upper and lower quartiles (data set 1a).

Animal	Type	Chemotherapeutic agent	Route of administration	Median	Q1	Q3
Mouse	Hydroxamate histone deacetylase inhibitors	JNJ-26481585	Subcutaneous	32.5	23.75	41.25
	Other	Salinomycin	Intraperitoneal	140	140	140
	Platinum compounds	Cisplatin	Hindpaw	0.004	0.002	0.022
			Intraperitoneal	20.5	8.25	23
			Subcutaneous	0.15	0.15	0.15
		Oxaliplatin	Hindpaw	0.04	0.04	0.04
			Intraperitoneal	10	3	30
			Intravenous	23	13.5	31
			Subcutaneous	0.04	0.04	0.04
	Proteasome inhibitors	Bortezomib	Intraperitoneal	0.75	0.425	1.95
			Intravenous	6.4	6.4	6.4
			Subcutaneous	12	12	18
	Taxanes	Paclitaxel	Intraperitoneal	10	5	16
			Intravenous	190	165	230
			Subcutaneous	10	10	10
			Tail vein	75	75	75
	Vinca alkaloids	Vincristine	Intraperitoneal	0.7	0.2	3.9
Rat	Binding of growth factor inhibitors	Suramin	Intravenous	25	17.5	37.5
	Other	Methotrexate	Subcutaneous	3.75	3.75	3.75
	Platinum compounds	Carboplatin	Intraperitoneal	112.5	101.25	123.75
		Cisplatin	Intraperitoneal	10	5	15
			Intravenous	2	2	2
			Subcutaneous	8	8	8
		Oxaliplatin	Intraperitoneal	16	8	32
			Intravenous	2	2	8
	Proteasome inhibitors	Bortezomib	Intraperitoneal	0.7	0.6	1
			Intravenous	4.8	4.2	4.8
	Taxanes	Docetaxel	Intravenous	25	17.5	32.5
			Tail vein	10	10	10
		Paclitaxel	Intraperitoneal	8	7.25	8.5
			Intravenous	24.5	9.75	33.25
			Tail vein	8	8	8
			Intrathecal^a^	6	3.1	13
		Nanoparticle albumin-bound paclitaxel	Intravenous	26.8	25.4	28.2
	Vinca alkaloids	Vincristine	Intraperitoneal	0.95	0.5	1
			Intravenous	0.435	0.2125	0.75
			Subcutaneous	0.42	0.42	0.42
			Tail	0.625	0.5625	0.6875

^a^ Drug doses are shown in mg/kg, except intrathecal paclitaxel, shown in ng.

The time to assessment did not account for a significant proportion of heterogeneity (τ^2^ = 2.55, I^2^ = 85.98%, *p* = 0.999).

In a post hoc analysis, we found that the strain of animal accounted for a significant proportion of the heterogeneity (Q = 269.58, df = 22, *p* < 0.01; Fig 4B). The most frequently reported strain was Sprague Dawley rats (−2.43 SD [95% CI −2.61 to −2.26], *n* = 437 comparisons).

#### Statistical power of different outcome measures

The number of animals required to achieve 80% power with a significance level of 0.05 varied substantially across the behavioural tests. For the most frequently reported behavioural tests—mechanical monofilaments, Randall-Selitto paw pressure test, electronic ‘von Frey’, acetone test/ethyl chloride spray, cold plate, and Plantar Test (Hargreave’s method)—we calculated the number of animals required in model and sham groups.

**Fig 5:**
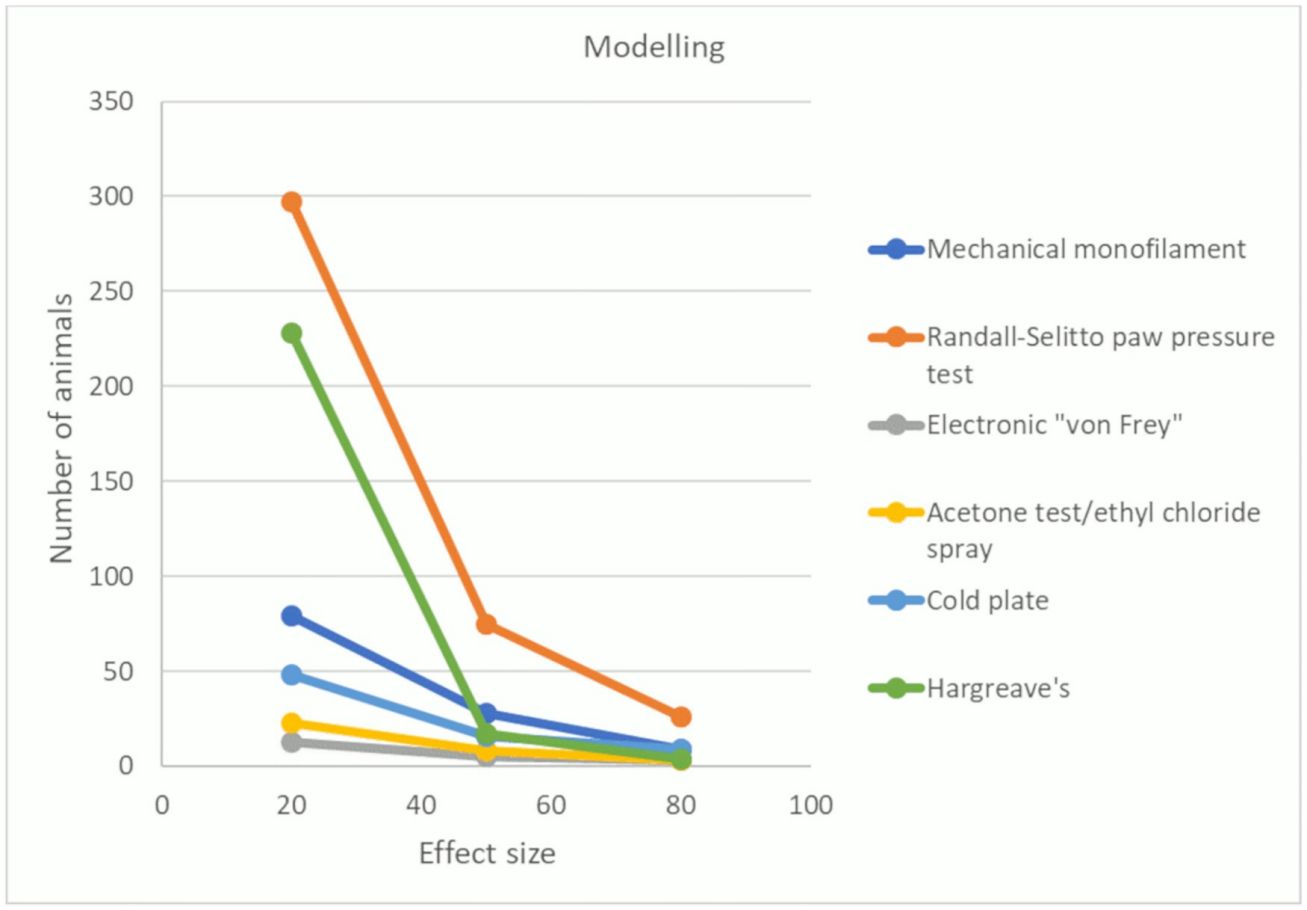
Power analysis for modelling experiments (data set 1a). Number of animals required per group to obtain 80% power with a significance level of 0.05 using mechanical monofilaments, Randall-Selitto paw pressure test, electronic ‘von Frey’, acetone test/ethyl chloride spray, cold plate, and Hargreave’s. Effect sizes calculated by SMD. SMD, standardised mean difference.

When both standardised mean difference (SMD) effect sizes and pooled SD were at the 50th percentile, the number of animals required ranged from 5 (electronic ‘von Frey’) to 75 per group (Randall-Sellito paw pressure test) (Fig 5). With an effect size at the 20th percentile and a variance at the 50th percentile, the number of animals required ranged from 13 (electronic ‘von Frey’) to 297 (Randall-Selitto paw pressure test) (Fig 5), demonstrating that some behavioural tests have less sensitivity to detect small effect sizes. The values for the 20th, 50th, and 80th percentiles of SMD effect sizes and SDs for each behavioural test are available on the OSF (https://doi.org/10.17605/OSF.IO/ZJEHY).

#### Risk of bias

**Fig 6:**
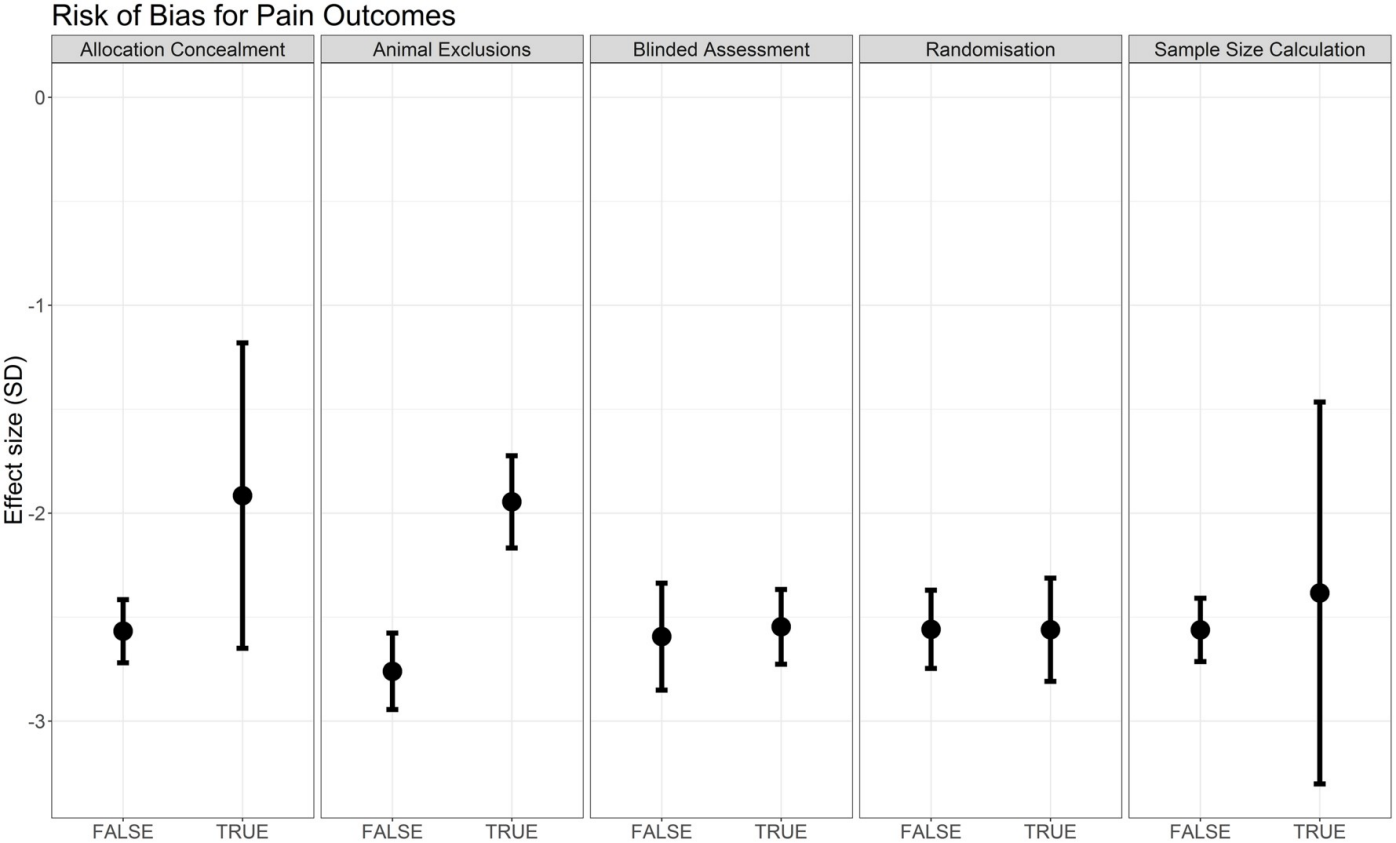
Effect sizes associated with measures to reduce risk of bias in modelling experiments using pain-related behavioural outcomes (data set 1a).

Reporting of blinded assessment of outcome (Q = 33.62, df = 1, *p* < 0.007) and animal exclusions (Q = 28.99, df = 1, *p* < 0.007) accounted for a significant proportion of the observed heterogeneity, although effect sizes for blinding are very similar between strata (in which strata refers to the subgroups of comparisons, i.e., blinded versus not blinded). Reporting of randomisation, allocation concealment, and sample size calculation did not account for a significant proportion of the observed heterogeneity (Fig 6); data table available on the OSF (https://doi.org/10.17605/OSF.IO/ZJEHY).

**Fig 7:**
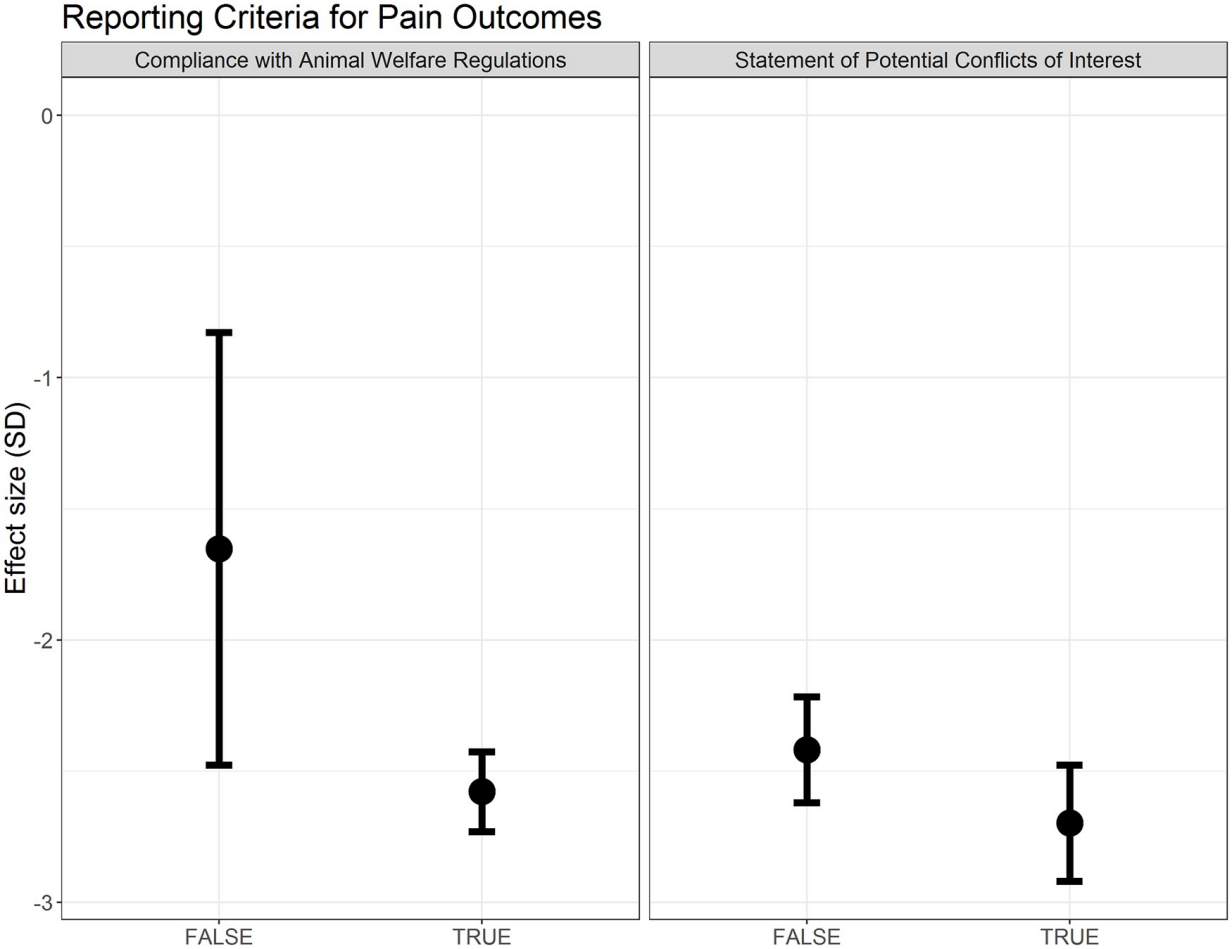
Effect sizes associated with reporting of compliance with animal welfare regulations and a statement of potential conflict of interests in modelling experiments using pain-related behavioural outcomes (data set 1a).

Compliance with animal welfare regulations accounted for a significant proportion of observed heterogeneity (Q = 19.44, df = 1, *p* < 0.007), although effect sizes are very similar between strata (Fig 7). Reporting of a conflict of interest statement did not account for a significant proportion of the heterogeneity (Fig 7).

#### Publication bias

**Fig 8:**
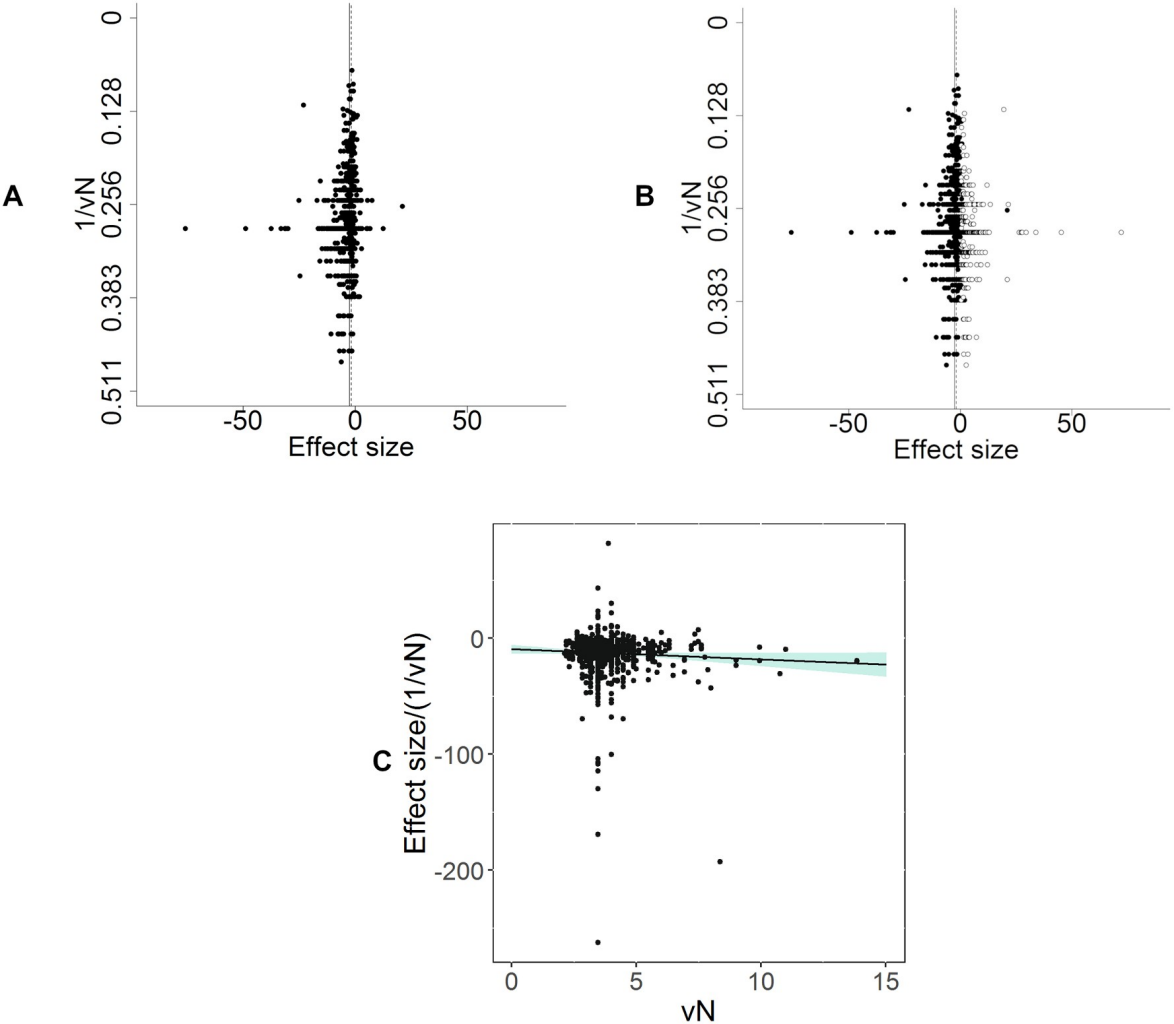
Assessment of publication bias in modelling experiments in which a pain-related outcome was used (data set 1a). (A) Visual inspection of the funnel plot suggests asymmetry. Filled circles represent reported experiments. Solid line represents global effect size, and dashed line represents adjusted global effect size. (B) Trim and fill analysis imputed theoretical missing studies (unfilled circles). Filled circles represent reported experiments. Solid line represents global effect size, and dashed line represents adjusted global effect size. (C) Egger’s regression indicated small study effects.

There were 1,123 individual comparisons (−2.58 SD [95% CI −2.72 to −2.45]). Visual inspection of funnel plots indicated asymmetry, suggesting missing studies (Fig 8A). Trim and fill analysis imputed 316 theoretical missing studies on the right-hand side of the funnel plot (Fig 8B). Inclusion of these theoretical missing studies decreased the estimate of modelling-induced pain-related behaviour by 30% to −1.82 SD (95% CI −1.97 to −1.68). Furthermore, Egger’s regression line and 95% CIs did not pass through the origin (*p* = 6.85 × 10^−7^), consistent with small study effects and again consistent with publication bias (Fig 8C).

### Animal studies modelling CIPN: Other behavioural outcomes (data set 1b)

In addition, as a secondary outcome, we abstracted data from modelling experiments using other behavioural outcomes (locomotor function, memory, reward behaviours, and attention). Administration of chemotherapeutic agents led to increased pain-related behaviours compared to sham controls (−0.75 [95% CI −1.04 to −0.47], *n* = 63 comparisons). Species did not account for a significant proportion of the heterogeneity (Q = 3.29, df = 1, *p* = 0.070), and therefore rats and mice were analysed together.

#### Study design

Type of outcome measure accounted for a significant proportion of the heterogeneity (Q = 25.44, df = 3, *p* < 0.01; all figures related to data set 1b are available in S4A Fig).

Chemotherapeutic agent accounted for a significant proportion of the heterogeneity (Q = 28.90, df = 7, *p* < 0.01; S4B Fig).

Sex (Q = 3.29, df = 1, *p* = 0.70) and time to assessment (τ^2^ = 0.63, I^2^ = 73.45%, *p* = 0.05) did not account for a significant proportion of the heterogeneity in modelling experiments using other behavioural outcomes.

A post hoc analysis found that strain accounted for a significant proportion of the heterogeneity (Q = 23.98, df = 6, *p* < 0.01; S4C Fig).

#### Risk of bias

Reporting of randomisation, blinding, allocation concealment, sample size calculation, or animal exclusions did not account for significant proportions of the heterogeneity (S5 Fig) nor did reporting of compliance with animal welfare regulations or a conflict of interest statement (S6 Fig).

#### Publication bias

There were 88 individual comparisons (−0.71 SD [95% CI −0.96 to −0.47]). Visual inspection of funnel plots indicated asymmetry, suggesting missing studies (S7A Fig). Trim and fill analysis imputed 21 theoretical missing studies on the right-hand side of the funnel plot (S7B Fig). Inclusion of these theoretical missing studies decreased the estimate of modelling-induced pain-related behaviour by 56% to −0.31 SD (95% CI −0.58 to −0.05). However, Egger’s regression was not consistent with small study effects (*p* = 0.293) (S7C Fig).

### Intervention experiments

#### Drug interventions in animal models of CIPN: Pain-related behavioural outcome measures (Data set 2a)

In CIPN intervention studies using pain-related behavioural outcome measures, administration of an intervention led to a 1.53 SD (95% CI 1.45–1.61) attenuation of pain-related behaviour compared to control (*n* = 1,360 comparisons, *p* < 0.007). Species did not account for a significant proportion of the heterogeneity (Q = 4.57, df = 1, *p* = 0.03), and so mouse and rat experiments were analysed together.

#### Study design

**Fig 9:**
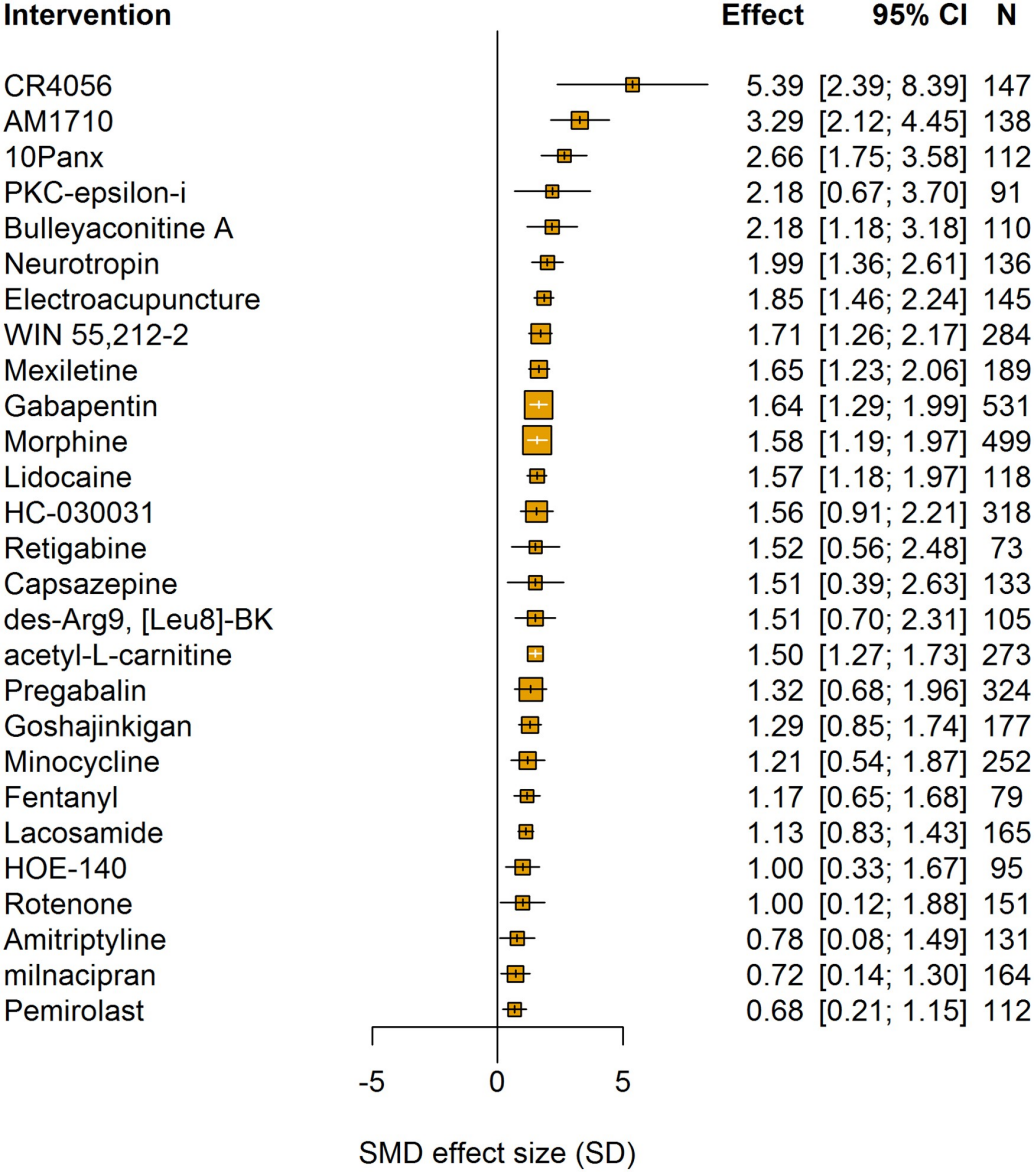
Intervention accounted for a significant proportion of the heterogeneity in intervention experiments using pain-related behavioural outcomes (data set 2a). Plot shows interventions with 10 or more comparisons. The size of the squares represents the number of nested comparisons that contribute to that data point, and the value *N* represents the number of animals that contribute to that data point.

The type of intervention accounted for a significant proportion of the heterogeneity (Q = 1,418.27, df = 304, *p* < 0.007). The most frequently tested interventions were morphine (*n* = 53 comparisons), gabapentin (*n* = 51 comparisons), and pregabalin (*n* = 35 comparisons) (Fig 9). No clear dose-response relationship was observed for any of these drugs, investigated by calculating the cumulative dose.

**Fig 10:**
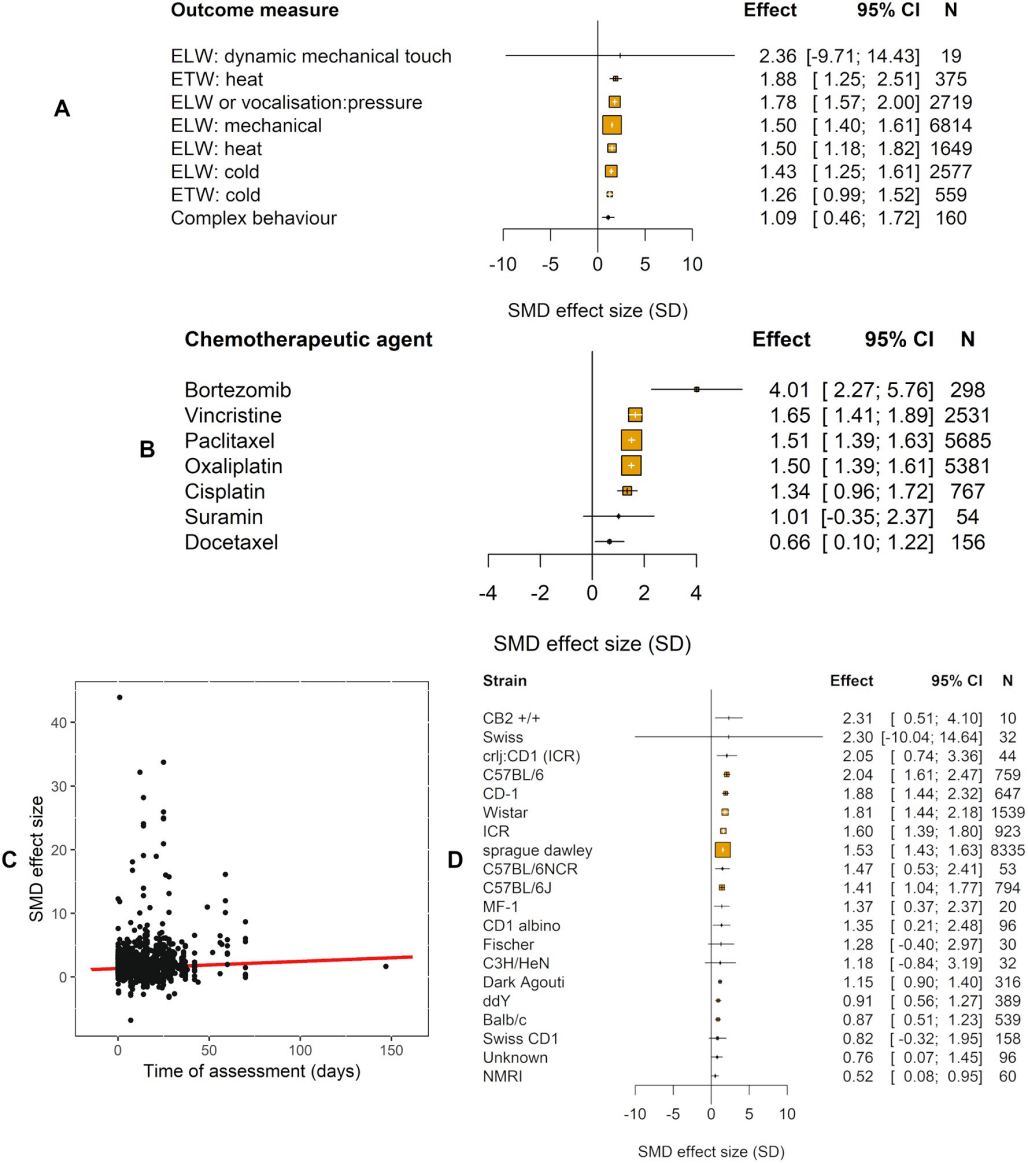
Impact of study design in intervention experiments using pain-related behavioural outcomes (data set 2a). The size of the squares represents the number of nested comparisons that contribute to that data point, and the value *N* represents the number of animals that contribute to that data point. (A) Outcome measure accounted for a significant proportion of the heterogeneity. (B) Chemotherapeutic agent accounted for a significant proportion of the heterogeneity. (C) Time of assessment accounted for a significant proportion of the heterogeneity. (D) Strain accounted for a significant proportion of the heterogeneity.

The type of pain-related outcome measure accounted for a significant proportion of the heterogeneity (Q = 24.36, df = 7, *p* < 0.007; Fig 10A).

In intervention studies, the chemotherapeutic agent used to induce the pain model accounted for a significant proportion of the heterogeneity (Q = 22.51, df = 6, *p* < 0.007; Fig 10B). The most frequently reported chemotherapeutic agents were paclitaxel (*n* = 520) and oxaliplatin (*n* = 480).

Sex of animal did not account for a significant proportion of the heterogeneity (Q = 3.27, df = 3, *p* = 0.35).

Time to assessment accounted for a significant proportion of the heterogeneity, with a longer interval associated with greater attenuation of pain-related behaviour (*p* < 0.007; Fig 10C). However, time of intervention administration did not account for a significant proportion of the heterogeneity (τ^2^ = 0.81, I^2^ = 57.51%, *p* = 0.5776).

In a post hoc analysis, we found that the strain of animal accounted for a significant proportion of the heterogeneity (Q = 120.25, df = 19, *p* < 0.007; Fig 10D). The most frequently reported were Sprague Dawley rats (*n* = 759).

#### Ranking drug efficacy

**Fig 11:**
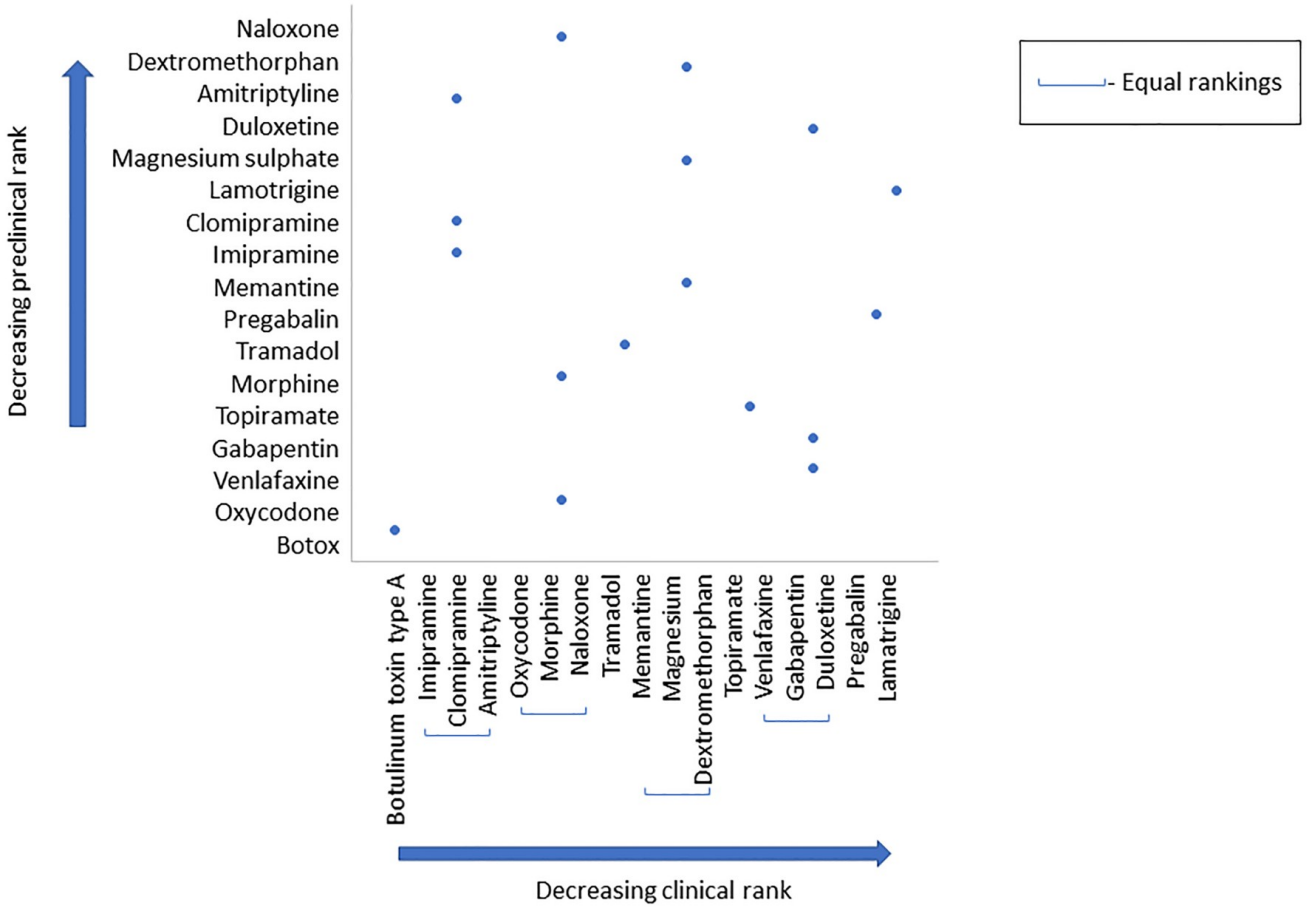
Rank order of clinical and preclinical drugs (data set 2a). A Spearman’s correlation was run to assess the relationship between clinical and preclinical rank of 17 drugs. There was no correlation between clinical and preclinical rank; r_s_ = −0.0099, *p* = 0.9699.

We performed a post hoc analysis in which we compared the ranking of drugs common between a clinical systematic review [18] and our review. A Spearman’s rank correlation coefficient found no correlation between clinical and preclinical rank (r_s_ = −0.0099, *p* = 0.9699; Fig 11).

#### Statistical power of different outcome measures

In intervention studies, the number of animals required to obtain 80% power with a significance level of 0.05 varied substantially across pain-related behavioural tests. For mechanical monofilaments, Randall-Selitto paw pressure test, electronic ‘von Frey’, acetone test/ethyl chloride spray, cold plate, and Plantar Test (Hargreave’s method), we calculated the number of animals required in intervention and control groups.

**Fig 12:**
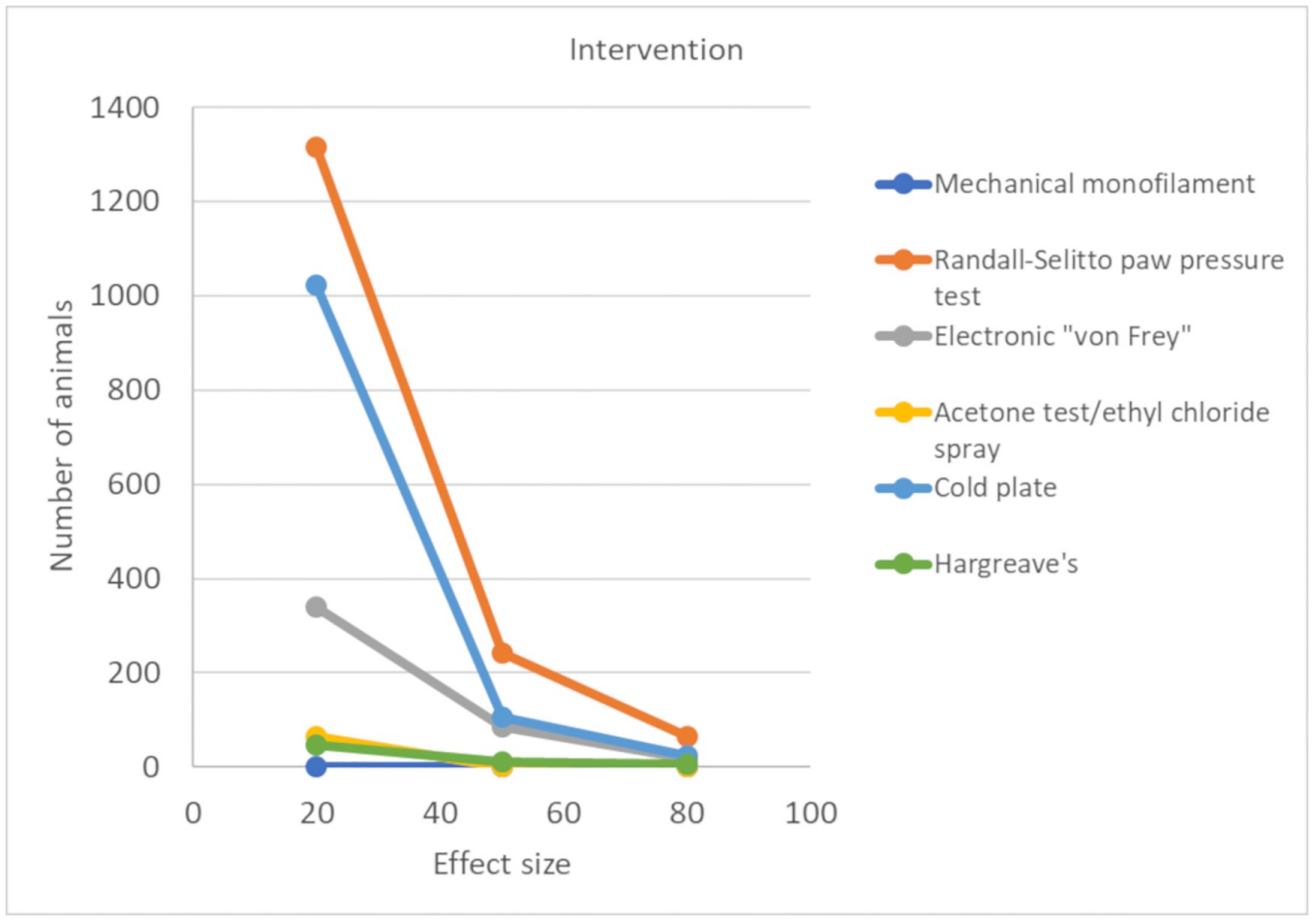
Power analysis for intervention experiments. Number of animals required to obtain 80% power with a significance level of 0.05 using mechanical monofilaments, Randall-Selitto paw pressure test, electronic ‘von Frey’, acetone test/ethyl chloride spray, cold plate, and Hargreave’s (data set 2a). Effect sizes calculated by SMD. SMD, standardised mean difference.

When both the SMD effect size and pooled SD were the 50% percentile, the number of animals required ranged from 8 (acetone test/ethyl chloride spray) to 242 (Randall-Selitto paw pressure test) (Fig 12). With an effect size at the 20th percentile and a variance at the 50th percentile, the number of animals required increased substantially, ranging from 46 (Hargreave’s) to 1,315 (Randall-Selitto paw pressure test). This again demonstrates that some behavioural tests have less sensitivity to detect small effect sizes. The values for the 20th, 50th, and 80th percentile of mean differences and pooled SDs for each behavioural test are provided on the OSF (https://doi.org/10.17605/OSF.IO/ZJEHY).

#### Risk of bias

**Fig 13:**
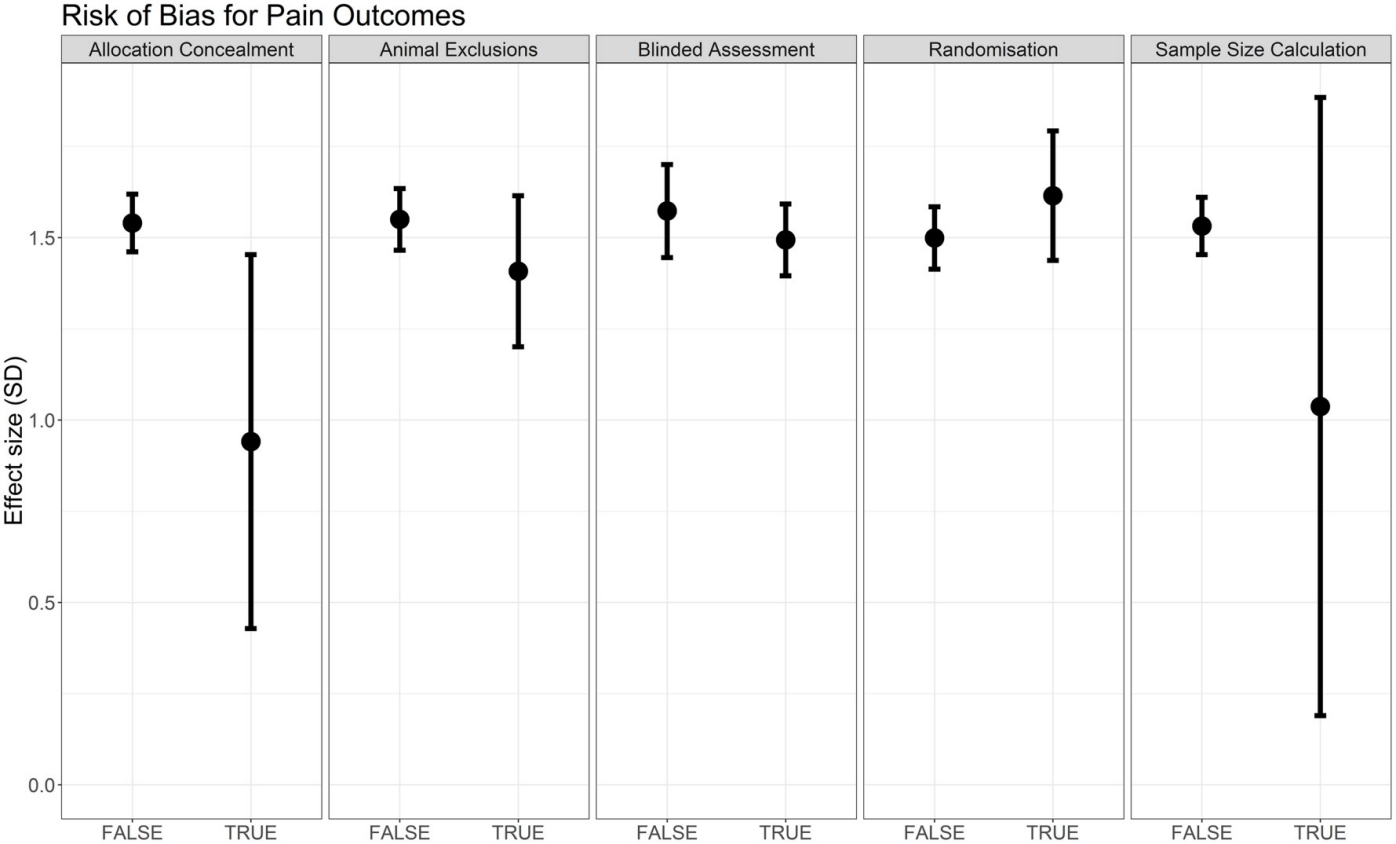
Effect sizes associated with measures to reduce risk of bias in intervention experiments using pain-related behavioural outcomes (data set 2a).

Reporting of allocation concealment, animal exclusions, and sample size calculations accounted for a significant proportion of the heterogeneity (Fig 13; data table available on the OSF; https://doi.org/10.17605/OSF.IO/ZJEHY), with studies that did not report these items giving greater estimates of effect. Reporting of randomisation and blinded assessment of outcome did not account for a significant proportion of the heterogeneity.

**Fig 14:**
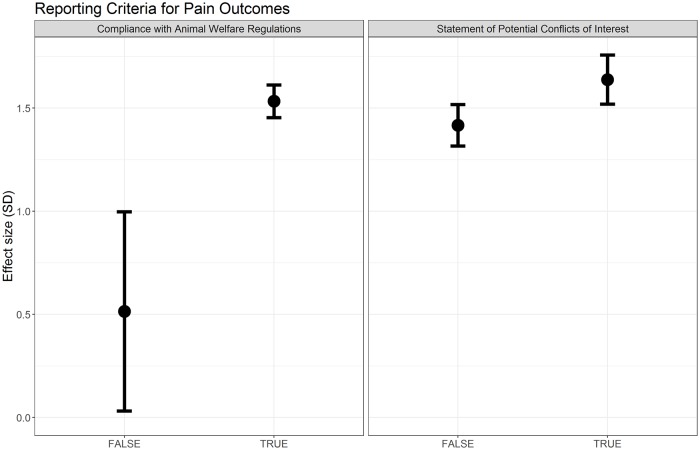
Effect sizes associated with reporting of compliance with animal welfare regulations and a statement of potential conflict of interests in intervention experiments using pain-related behavioural outcomes (data set 2a).

Both reporting of compliance with animal welfare regulations (Q = 8.86, df = 1, *p* < 0.007) and reporting of a conflict of interest statement (Q = 8.28, df = 1, *p* < 0.007) accounted for a significant proportion of the heterogeneity (Fig 14). Failure to report this information was associated with smaller estimates of effect.

#### Publication bias

**Fig 15:**
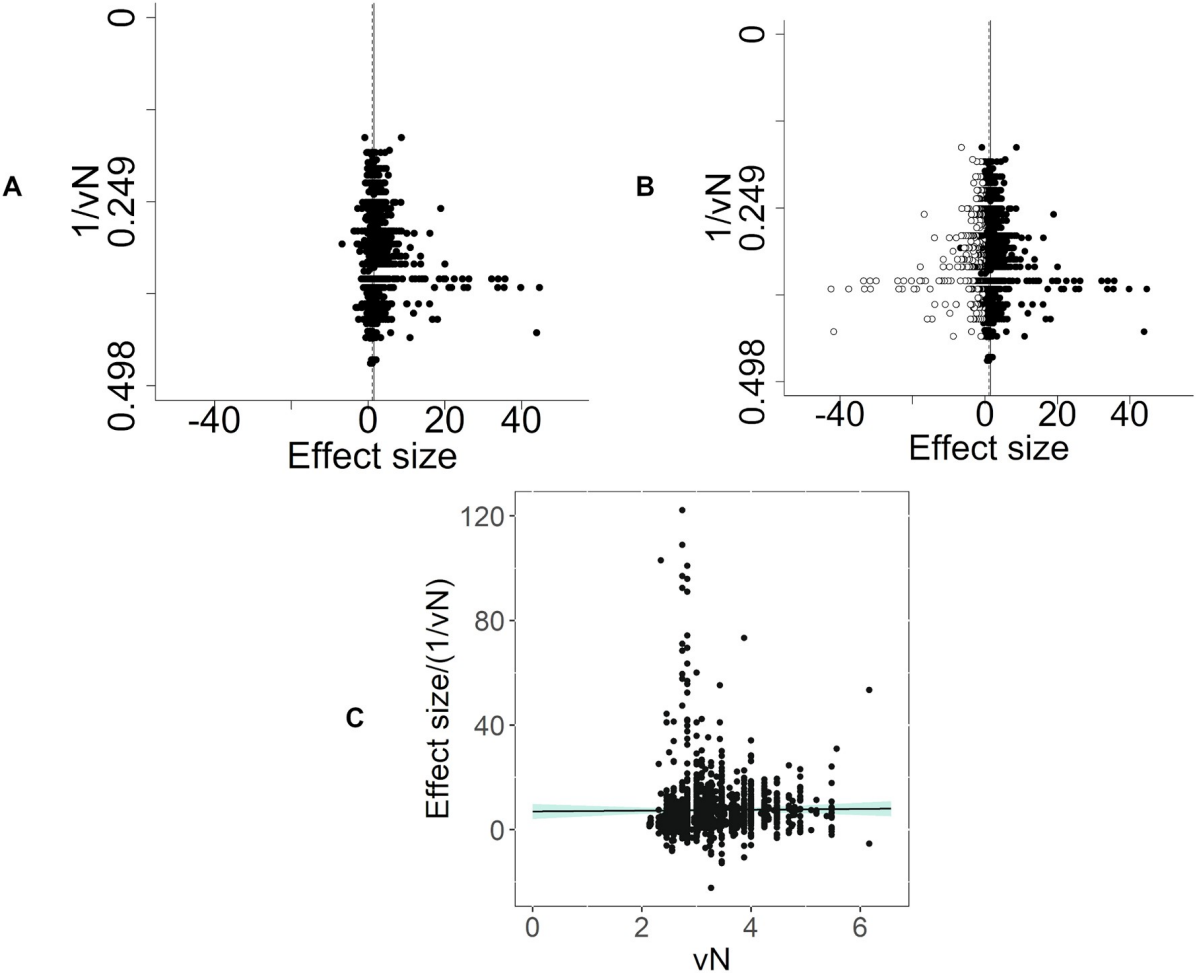
Intervention experiments in which a pain-related outcome was used (data set 2a). (A) Visual inspection of the funnel plot suggests asymmetry. Filled circles represent reported experiments. Solid line represents global effect size, and dashed line represents adjusted global effect size. (B) Trim and fill analysis imputed theoretical missing studies (unfilled circles). Filled circles represent reported experiments. Solid line represents global effect size, and dashed line represents adjusted global effect size. (C) Egger’s regression indicated small study effects. vN, square root of N.

There were 1,513 individual comparisons (1.52 SD [95% CI 1.44–1.59]). Visual inspection of funnel plots indicated asymmetry, suggesting missing studies (Fig 15A). Trim and fill analysis imputed 389 theoretical missing studies on the left-hand side of the funnel plot (Fig 15B). The inclusion of these theoretical missing studies decreased the estimate of intervention effects by 28% to 1.09 SD (95% CI 1.01–1.16). Furthermore, Egger’s regression was consistent with small study effects (*p* = 2.17 × 10^−6^), suggesting funnel plot asymmetry (Fig 15C).

### Drug interventions in animal models of CIPN: Other behavioural outcomes (data set 2b)

In intervention studies using other behavioural outcomes, administration of interventions led to improvement in other behaviours compared to controls (0.69 SD [95% CI 0.37–1.0], *n* = 37 comparisons). Species did not account for a significant proportion of the heterogeneity (Q = 0.75, df = 1, *p* = 0.39).

#### Study design

Two outcome measures were used, and this accounted for a significant proportion of the heterogeneity. We observed greater improvement in reward-related behaviours compared with locomotor function (1.61 SD [95% CI 1.13–2.09], *n* = 6) comparisons versus (0.52 [95% CI 0.20–0.85], *n* = 31 comparisons [Q = 13.70, df = 1, *p* < 0.007]; S8A Fig).

The type of intervention accounted for a significant proportion of the heterogeneity (Q = 51.82, df = 19, *p* < 0.007; S9 Fig).

Chemotherapeutic agent (Q = 2.21, df = 1, *p* = 0.137), sex (Q = 9.67, df = 2, *p* = 0.008), time to assessment (τ^2^ = 0.37, I^2^ = 54.21%, *p* = 0.398), and time of intervention administration (τ^2^ = 0.32, I^2^ = 52.37%, *p* = 0.331) did not account for a significant proportion of the heterogeneity.

In a post hoc analysis, we found that strain accounted for a significant proportion of the heterogeneity (Q = 16.18, df = 3, *p* < 0.007; S8B Fig).

#### Risk of bias

Blinded assessment of outcome accounted for a significant proportion of the heterogeneity (Q = 8.11, df = 1, *p* < 0.007) (S10 Fig). Reporting of randomisation or animal exclusions did not account for a significant proportion of the heterogeneity. No studies in this data set reported allocation concealment or the use of a sample size calculation. Reporting of a conflict of interest statement did not account for a significant proportion of the heterogeneity (S11 Fig). All studies in this data set reported compliance with animal welfare regulations.

#### Publication bias

There were 43 individual comparisons (0.70 SD [95% CI 0.41–0.99]). Visual inspection of funnel plots did not indicate asymmetry, suggesting no missing studies. Trim and fill analysis estimated no theoretical missing studies. Furthermore, Egger’s regression was not consistent with small study effects (*p* = 0.352), suggesting funnel plot symmetry.

### Animal husbandry

The reporting of details of animal husbandry was low across all included studies (S1 Table). No study reported whether different species were housed in the same room.

## Discussion

Our systematic review and meta-analysis includes data from 337 publications describing animal models of CIPN. We demonstrate in modelling experiments that administration of a chemotherapeutic agent compared with sham controls leads to an increase in pain-related behaviours, and in intervention studies, drug administration attenuates pain-related behaviours.

Animal models of CIPN are used to elucidate the pathophysiology of the condition and to develop potential therapies. Our purpose here was to synthesise and summarise the entirety of the animal model CIPN literature primarily to make it accessible to scientists interested in the field and to provide them with data from which they can efficiently select optimal models to suit their experimental aims and to plan their experiments to a high level of rigour (e.g., suitably informed sample size calculations).

Here, we show—in 2 cohorts of primary studies, those modelling CIPN compared to sham controls and those testing the effect of intervention in animal models of CIPN—that there are some limitations in their experimental design. Our primary focus was on pain-related behaviours. Most studies used only male animals (84%) and evoked limb withdrawal to mechanical stimuli. Reporting of measures to reduce risk of bias was moderate. Our indicative power calculations allow the ranking of the most commonly reported pain-related behavioural tests and suggest that the Randall-Sellito paw pressure test may be the least sensitive to detect small effect sizes. Our analyses also indicate likely publication bias and estimate an average of a 30% relative overestimation on reported results. This empirical evidence and our suggestions may generate discussion to guide the design of future studies and the importance of disseminating experimental findings irrespective of their direction of effect.

In undertaking this review, we observed increasing rates of publications describing primary studies of animal models of CIPN and that the accrual rate of relevant publications increased by 89% in 3 years. This presents technical challenges in synthesising a large data set in a timely manner. We were able to demonstrate the feasibility of using machine learning to facilitate screening for inclusion in systematic reviews of preclinical studies.

### External validity of studies using animal models of CIPN

#### Misalignment between animal models and the clinical population

Most identified studies (285 out of 341 [84%]) used only male animals to model CIPN. In the clinic, the chemotherapeutic agents included in this analysis are frequently used to treat female cancer patients (e.g., ovarian or breast carcinoma), thus reducing the generalisability of the findings from these models to the clinical population. Sex only accounted for a significant proportion of the heterogeneity in the modelling of painful neuropathy in which pain-related behaviours were measured. It is likely that the paucity of female animals limits our ability to ascertain with sufficient power the impact of sex on models of CIPN in other contexts. Karp and colleagues have demonstrated that a large number of mammalian phenotypic traits are sexually dimorphic [19], and in line with National Institutes of Health policy [20], we advocate for the use of female animals in addition to males.

We also have concerns about the clinical relevance of the time courses frequently studied in animals. Acute CIPN is estimated to affect 68% of patients within the first month of chemotherapy cessation, 60% at 3 months, and 30% of patients at 6 months [5]. However, chronic CIPN has been observed in four-fifths of patients exposed to taxane [21] or oxaliplatin [22] approximately 2 years after treatment. A long-term study showed that oxaliplatin treatment was associated with CIPN at 6-year follow-up [23]. Therefore, the short duration of these models of CIPN identified in this systematic review likely model the acute phase. Of those publications in which study duration (the time between the first administration of chemotherapeutic agent and the time when animals were euthanised) was reported (39 of 341 publications), the median duration was only 21 days (16–28 IQR). Furthermore, the median time to outcome assessment in our modelling data set (the time in which there was the biggest difference between CIPN model and sham animals) was 14 days (7–25 IQR). The median time to outcome assessment in our intervention data set was also 14 days (7–22 IQR), indicating that the time in which the drug interventions are most effective is when the models show the largest modelling effect.

#### Misalignment between preclinical and clinical outcome measures

The most frequent behaviours reported in animal models of CIPN are manifestations of gain in sensory function; hypersensitivity in paw withdrawal evoked by mechanical stimuli was the assay most often employed. A review of studies reporting preclinical models of pain published in the journal *Pain* between 2000 and 2004 also found that the most frequently reported pain-related behaviours were such reflex withdrawal responses [24]. This contrasts with chronic CIPN in clinical practice, in which the predominant clinical sensory phenotype of these patients is one of sensory loss [4,25], and this may compromise the clinical relevance of these models for chronic CIPN; however, they may have more relevance to acute CIPN.

One approach to addressing this misalignment between outcome measures used to assess pain in patients in clinical trials and those frequently reported in animal models of CIPN would be the development of sensory profiling for rodent models of neuropathy that better reflect the clinical picture [25].

### Internal validity of studies using animal models of CIPN

There was moderate reporting of measures to reduce risk of bias. Our subgroup analyses did not consistently identify that the reporting of these measures had an impact on experimental findings. It may be that there was insufficient power to test for these associations because of the small number of studies reporting these factors or that there is indeed no association. We also are only able to test the reporting of these measures to reduce risk of bias, and these may differ according to the actual use of these measures in the design, conduct, and analysis of a study. The details of methods used to implement randomisation and blinding and the methods and assumptions for sample size calculation were rarely reported. Despite the inconsistency of our findings, there is substantial empirical evidence of numerous research domains that these details are important to understand the validity of the procedures used [17], noting that one of the included studies reported that randomisation was achieved by selecting animals at random from the cage. If methods and assumptions were reported, this would allow assessment of the quality of these procedures that report using tools such as those used in clinical systematic reviews [26] and allow for more robust assessments of their impact on research findings.

Statistical modelling and meta-analysis have demonstrated that the exclusion of animals can distort true effects; even random loss of samples decreased statistical power, but if the exclusion is not random, this can dramatically increase the probability of false positive results [27]. It has been shown in other research fields that treatment efficacy is lower in studies that report measures to reduce risk of bias [13,28–30].

### Publication bias

Our assessment of publication bias finds evidence to suggest that global effect sizes are substantially overstated in all data sets except the smallest (and this is likely due to reduced power to detect publication bias with only 37 studies). We observed relative overstatements in effect sizes that ranged from 28% to 56%. Publication bias is a prevailing problem in preclinical research, in which neutral or negative studies are less likely to be published than positive studies [31]. One potential reason for this is the high competition for academic promotion and funding, and few incentives to publish findings from studies in which the null hypothesis was not disproved. Initiatives such as Registered Reports provide one mechanism to support the publication of well-designed, thoroughly executed, and well-reported studies asking important questions regardless of the results.

### Optimising experimental design

Experimental design of in vivo CIPN studies could be optimised by adopting measures to reduce risk of bias, such as using sample size calculations to ensure that experiments are appropriately powered. It is also important to use a model that best represents the clinical population of interest, for example, using both female and male animals. To help further address the issue of publication bias, we suggest that researchers make available prespecified protocols for confirmatory preclinical studies and publish all results. Others have shown that external validity may be increased by using multicentre studies to create more heterogeneous study samples, for example, by introducing variations in the animal genetics and environmental conditions (housing and husbandry) between laboratories, an approach that may be useful in pain modelling.

One approach that would help optimise experimental design is to use the Experimental Design Assistant (EDA; https://eda.nc3rs.org.uk/), a free resource developed by the NC3Rs, whereby researchers create a record of their experimental design [32]. The output from the EDA could then be uploaded to the OSF as a record for transparency.

### Reduction

There are opportunities to reduce waste and maximise the information gained from in vivo models of pain studies. This would require open and transparent reporting of results. For example, for complex behaviours, the online dissemination of individual animal video files [33] would allow reanalysis for further behaviours not reported in the original publication. It is interesting to note that although the open field was used in studies included in this systematic review, none of the included studies reported thigmotaxis, an outcome measure reported in other preclinical pain research. Sharing open field video files would allow this outcome to be assessed from previously conducted experiments. Our exemplar power calculations of the most frequently reported behavioural outcome measures highlight the substantial variability in the statistical performance of different outcome measures. Using these results, it is possible to rank the different pain-related behavioural tests according to how many animals are required per group as effect size or SD increases or decreases. This allows researchers to evaluate the sensitivity of their estimates of numbers required compared with variations in the effect sizes or variance achieved. Along with other factors, such as clinical relevance, these results can inform the choice of outcome measure in study design by allowing researchers to select outcome measures that require fewer animals.

The results of our systematic review show increasing rates of publications of experiments using animal models of CIPN. Between the initial search in 2012 and the updated search in 2015, the number of relevant publications increased by 89%. The high publication accrual rate is not unique to this field but is the case across clinical [34] and preclinical research; this makes it challenging for researchers and consumers of research to keep up to date with the literature in their field. This systematic review of preclinical models of pain uses machine learning and text mining and demonstrates the usefulness of these automation tools in this field.

### Limitations

Conducting a systematic review is time and resource intensive, and the rate of publication of new primary research means that systematic reviews rapidly become outdated. This review is limited because the most recent information included was identified in November 2015. We plan that the present systematic review form a ‘baseline’ systematic review, which can be updated and developed into a living systematic review, i.e., one that is continually updated as new evidence is published [35]. An important secondary output of this review is the advances made in the use of machine learning to facilitate the automation of systematic reviews of preclinical studies. As new online platforms and tools for machine learning and automation become available, preclinical living systematic reviews become more feasible [36]. Guidelines for living systematic reviews [36] and the use of automation tools [37] have recently been published, and Cochrane has also launched pilot living systematic reviews [38,39].

The machine-learning algorithm based on our initial screening had a high sensitivity (97%) and medium specificity (67%). High sensitivity has a low risk of missing relevant literature. An algorithm with lower specificity is more likely to falsely identify studies for inclusion (i.e., false positives). As a result, during data abstraction, the 2 independent human screeners excluded many studies identified by the machine for inclusion. We believe that this balance between sensitivity and specificity was appropriate because this reduced the risk of missing relevant studies.

A further possible limitation of our study is that we chose to extract behavioural data at the time point at which there was the largest difference between model and sham control animals or treatment and control animals. This time point was chosen to capture information on intervention effects regardless of their half-life. This limits what we can infer regarding the mismatch between timings, but we did also capture information on the first administration of intervention (relative to induction of the model) and the last administration. Future studies may use area under the curve approaches to capture response to model induction or drug intervention, but this was not possible for this large data set. There are tools under development for automation of data extraction, which may assist progress in this area [40].

In our meta-analysis, we grouped together the behavioural outcome measures that measure the same underlying biology. For example, in the case of experiments that reported using the grip test, 5 studies reported that the test was used to measure grip strength, and 1 reported that the test was used to measure muscle hypersensitivity [41]. For this reason, in our analysis, we grouped all grip test outcome measures together as a non–pain-related behavioural outcome measures. It is possible that the same tests or similar tests could be used and the same measurements reported as different outcomes; one test may also measure multiple facets of underlying biology. This is one of the challenges when analysing published data, and principle components analyses of large data sets such as these may help identify latent domains of behavioural outcome.

We only included studies in which the intervention drug was administered after or at the same time as the chemotherapeutic agent. Future literature reviews may consider drug interventions given before chemotherapeutic agents to determine whether prophylaxis can effectively prevent CIPN.

Unfortunately, the reporting of measures to reduce risk of bias was moderate in the studies included in this systematic review, which limits what we can infer from the results. We hope this review will highlight this issue in in vivo modelling of CIPN. Systematic review of animal experiments in other research areas has revealed low reporting of these measures and the negative impact of failure to report these measures across in vivo domains as diverse as modelling of stroke, intracerebral haemorrhage, Parkinson’s disease, multiple sclerosis, and bone cancer pain [15,29,30,42–44]. This has driven change, influencing the development of reporting guidelines [45], pain modelling specific guidelines [46], and the editorial policy of Nature Publishing Group [47]. However, requesting that submitting authors complete a reporting guideline without any other intervention is not associated with improved reporting [48]. After an initial review on the efficacy of interleukin-1 receptor antagonist in animal models of stroke highlighted low reporting of measures to reduce risk of bias [49], a subsequent review identified increased reporting of these measures [15], increasing the validity and reliability of these results. We hope that there will be a similar improvement in studies reporting the use of animal models of CIPN. We propose that if more studies implement and report measures to reduce the risk of bias, it will be possible to use a GRADE-type analysis to rate the certainty of the evidence of animal studies [50]. At present, any such approach is likely to lead to the majority of evidence being downgraded to the extent that no firm conclusions can be drawn. The measures to reduce risk of bias that we have assessed are largely derived from what is known to be important in clinical trials, and the extent to which these measures are important in animal studies has yet to be fully elucidated. However, reporting of these measures allows users of research to make informed judgments about the fidelity of the findings presented. Equally, it may be that there are other measures that are important in animal studies that we have not considered.

A recent study from our group has suggested that using SMD estimates of effect sizes with stratified meta-analysis has a moderate statistical power to detect the effect of a variable of interest when there are 200 included studies but that the false positive rate is low. This means that although we may not have sufficient power to detect an effect, we can have confidence that any significant results observed are likely to be true [51].

### Conclusions

This systematic review and meta-analysis provides a comprehensive summary of the in vivo modelling of CIPN. The data herein can be used to inform robust experimental design of future studies. We have identified some areas in which the internal and external validity of preclinical CIPN studies may be increased; using both sexes of animals in the modelling of CIPN and ensuring outcome measures align with those most relevant in the clinic will likely improve external validity. Measures to reduce risk of bias should be employed to increase the internal validity of studies. Power analysis calculations illustrate the variation in group size under different conditions and between different behavioural tests and can be used to inform outcome measure choice in study design.

## Materials and methods

This review forms part of a larger review of all in vivo models of painful neuropathy, and the full protocol is available at www.dcn.ed.ac.uk/camarades/research.html#protocols. Our review protocol predates the opening of the PROSPERO registry to reviews of in vivo preclinical data. Methods used were prespecified in the study protocol.

### Search strategy

In September 2012, we systematically searched 5 online databases (PubMed, Web of Science, Biosis Citation Index, Biosis Previews, and Embase) with no language restrictions to identify publications reporting in vivo modelling of CIPN that reported a pain-related behavioural outcome measure. The search terms used for each database are detailed in S1 File. Search results were limited to animal studies using search filters [52,53]. Because we anticipated a high accrual rate of new publications, we ran an updated search in November 2015 and used machine learning and text mining to reduce the screening for inclusion workload. This updated search included 4 online databases (PubMed, Web of Science, Biosis Citation Index, and Embase) and used an updated animal filter [54]. Biosis Previews was no longer available.

### Machine learning and text mining

We used machine learning to facilitate the screening of publications reporting animal models of CIPN and improve accuracy of the screening process [55]. The screening stage of a systematic review involves ‘including’ or ‘excluding’ publications identified in the search based on their title and abstract, and this was performed by 2 independent reviewers. The publications from our initial search (with ‘include’/‘exclude’ decisions based on initial dual screening and differences reconciled by a third reviewer; inter-reviewer agreement Kappa = 0.95, standard error [SE] = 0.002) were used as a training set for machine learning approaches applied to the updated search.

Five machine learning groups participated, and 13 classifiers were created and applied to the updated search (validation set) [16]. We manually screened 10% of the updated publications (*n* = 1,188) and used this to assess the performance of these classifiers using measures of sensitivity, specificity, and precision as described by O’Mara-Eves and colleagues (2015) [56]. The reconciled decision of the human reviewers was considered the gold standard. We chose cut-off points such that the sensitivity of each classifier reached 0.95 and measured the resulting specificity and precision to choose the classifier that performed best for our data set.

To test the performance of the classifiers in the validation set, we used a random number generator to select a 10% random sample, and 2 independent investigators checked these for inclusion or exclusion. From the included studies in the updated search, we used text mining to identify studies reporting animal models of CIPN by searching for specific chemotherapy terms within the title and abstract of the identified publications; the inclusion of these studies was then verified by 2 independent reviewers.

### Inclusion and exclusion criteria

We included controlled studies using pain-related behavioural outcome measures that either characterised models of neuropathy induced by chemotherapeutic agents or tested the effect of a drug intervention in such models (Fig 2). We required that studies report the number of animals per group, the mean, and a measure of variance (either the standard error of the mean [SEM] or the SD).

We excluded studies that administered the drug intervention before model induction, administered co-treatments, used transgenic models, or used in vitro models.

### Measures to reduce risk of bias

We assessed the risk of bias of included studies by recording the reporting of 5 measures to reduce risk of bias at the study level: blinded assessment of outcome, random allocation to group, allocation concealment, reporting of animal exclusions, and a sample size calculation [57].

We also assessed the reporting of a statement of potential conflicts of interest and of compliance with animal welfare regulations [57,58].

### Data abstraction

Data were abstracted to the CAMARADES Data Manager (Microsoft Access, Redmond, WA). For all included studies, we included details of publication (Table 5), animal husbandry, model, intervention, and other experiment details (Table 6). Outcome data presented graphically were abstracted using digital ruler software (Universal Desktop Ruler, AVPSoft.com or Adobe ruler) to determine values. When multiple time points were presented, we abstracted the time point that showed the greatest difference between model and control groups, or the greatest difference between treatment and control groups. If the type of variance (e.g., SEM or SD) was not reported, we characterised the variance as SEM because this is a more conservative approach in meta-analysis, in which studies are weighted in part by the inverse of the observed variance. All data were abstracted by 2 independent reviewers.

**Table 5:** Publication level data abstracted from each publication.

Publication level		
Metadata	Risk of bias	Reporting quality
First author Corresponding author Year	Random allocation Allocation concealment Blinded assessment Sample size calculation Animal exclusions	Compliance with animal welfare regulations Statement of potential conflict of interests

**Table 6:** Experiment level data abstracted from each publication.

Experiment level				
Animal	Husbandry	Model	Intervention	Outcome measure
Species Strain Sex Supplier Country of supplier	Type of diet Cage rack ventilation Type of bedding Enrichment Habituation time prestudy Cage cleaning frequency Number per cage Shared cages between CIPN and sham animals Number of hours in light cycle Room temperature Humidity Food availability Different species housed in same room	Chemotherapeutic agent Dose Number of doses Route of administration Model duration Weight Age	Time of drug administration Dose Route of administration	Outcome measure type Units Larger values indicates better/worse Number of groups served by control group Time of assessment Number of animals per group Mean outcome Variance Number of treatment groups per control Number of animal exclusions and reason

**Abbreviation**: CIPN, chemotherapy-induced peripheral neuropathy.

### Data reconciliation

Publication and outcome level data abstracted by 2 independent reviewers were compared, and any discrepancies were reconciled. For outcome data, SMD effect sizes of individual comparisons were calculated for each reviewer’s extracted data, and when these differed by ≥10%, they were identified for reconciliation. When individual comparisons differed by <10%, we took a mean of the 2 effect sizes and of the variance measure.

### Data analysis

We separated the data according to those reporting the modelling of CIPN only and those testing the effect of an intervention in a model of CIPN. We analysed all the behavioural outcome measures reported. Behavioural outcome measures were separately considered as ‘pain-related’ or ‘other (non–pain related)’ behavioural outcome measures (Fig 2). This resulted in 4 data sets: (1) animal studies modelling CIPN: pain-related behavioural outcome measures (data set 1a), (2) animal studies modelling CIPN: other behavioural outcomes (data set 1b), (3) drug interventions in animal models of CIPN: pain-related behavioural outcome measures (data set 2a), and (4) drug interventions in animal models of CIPN: other behavioural outcomes (data set 2b). Data from individual experiments were extracted from each publication, and these are reported as ‘individual comparisons’.

For each individual comparison, we calculated an SMD effect size. When more than one relevant behaviour was reported in the same cohort of animals, these individual comparisons were aggregated (‘nested comparisons’; Fig 2) by behavioural subtype, determined by the site of stimulus application (e.g., limb or tail) and the modality of the stimulus used (e.g., mechanical or heat). Fixed-effects meta-analysis was used to give a summary estimate of these effects in each cohort. Cohort-level effect sizes were then pooled using a random-effects meta-analysis with restricted maximum-likelihood estimation of heterogeneity, in which heterogeneity refers to the variation in study outcomes between studies. When a single control group served multiple comparator (model or treatment) groups, their contribution was adjusted by dividing the number of animals in the control group by the number of comparator groups served. The Hartung and Knapp method was used to adjust test statistics and confidence intervals; this calculates the confidence intervals using the following formula: effect size + t(0.975,k − 1) × SE. Results are presented in SMD units along with the 95% confidence intervals.

To provide empirical evidence to inform experimental design and refine modelling of CIPN, we assessed the extent to which predefined study design and study risk of bias characteristics explained observed heterogeneity. We used stratified meta-analysis for categorical variables and metaregression for continuous variables. The purpose of these subgroup analyses is to observe whether studies grouped together describing a similar characteristic (e.g., all studies using male animals versus all studies using female animals) differ in their overall estimates of effects. Such analyses provide empirical evidence of the impact of study design choices and are useful to design future experiments. The study design factors assessed using stratified meta-analysis were animal sex and species, therapeutic intervention, therapeutic intervention dose, methods to induce the model including the chemotherapeutic agent, and type of outcome measure. Because drug dose and route of administration are largely important in the context of the intervention being used, we did not assess the impact of dose or route of administration across different chemotherapeutic agents or drug interventions. We specified a priori that if species accounted for a significant proportion of heterogeneity, we would analyse the effect of study design factors on each species separately. If not, then all data would be analysed together. We also assessed the impact of reporting of measures to reduce bias. We used metaregression to assess the impact of time to assessment (defined as the interval between first administration of chemotherapeutic agent and outcome measurement) and time to intervention administration (defined as the interval between first administration of chemotherapeutic agent and administration of intervention). We used a meta-analysis online platform (code available here: https://github.com/qianyingw/meta-analysis-app) to perform all meta-analyses.

We applied a Bonferroni-Holmes correction for multiple testing that resulted in critical thresholds for significance as follows: in modelling experiments, *p* < 0.01 for study design features and *p* < 0.007 for reporting of measures to reduce risk of bias and measures of reporting; in intervention experiments, *p* < 0.007 for study design features, and *p* < 0.007 for reporting of measures to reduce risk of bias and measures of reporting.

### Power analysis of in vivo modelling

To guide sample size estimation for future studies, we performed power calculations for the 6 most frequently reported behavioural tests. To do this, we separately ranked the observed SMD effect size and the pooled SD and, for each, identified the 20th, 50th, and 80th percentile. We then used these values to calculate the number of animals required in 9 hypothetical treatment and control groups. Calculations were based on the two-sample two-sided *t* test, with 80% power and an alpha value of 0.05.

### Publication bias

We assessed for potential publication bias by assessing the asymmetry of funnel plots using visual inspection and Egger’s regression [59]. We assessed for the impact of publication bias using Duval and Tweedie’s trim and fill analysis [60,61]. We performed these assessments in 4 data sets separately and used individual comparisons rather than summary estimates for each cohort (Fig 1).

### Comparison of intervention efficacy with that observed in human studies

In a clinical systematic review of neuropathic pain [18], selected analgesic agents had been ranked according to their efficacy, as measured by Number Needed to Treat (NNT) for 50% pain relief. If preclinical studies included in this review reported use of these agents or their analogues, we ranked the interventions according to their SMD effect size for attenuation of pain-related behaviour. We then assessed the correlation between clinical and preclinical rank using Spearman’s rank correlation coefficient.

## Supporting information

S1 Table Reporting of animal husbandry details. The median habituation time was 7 days (7–7 IQR). The median number of animals per cage was 4 (2.5–4.5). Reporting of mixed housing with shams was always ‘Not mixed’. Room temperature 22 °C (22 °C–23 °C IQR). Humidity 55 (53.75–55 IQR).
(DOCX)

S1 Fig Number of included publications published each year. (TIF)

S2 Fig Cumulative meta-analysis of (A) effect sizes and (B) tau^2^ estimates, ordered by year of publication. (TIF)

S3 Fig Tree plot of prevalence of interventions. A total of 306 different interventions reported.
(TIF)

S4 Fig Impact of study design in modelling experiments using other behavioural outcomes. The size of the squares represents the number of nested comparisons that contribute to that data point, and the value *N* represents the number of animals that contribute to that data point. (A) Outcome measure accounted for a significant proportion of the heterogeneity. (B) Chemotherapeutic agent accounted for a significant proportion of the heterogeneity. (C) Strain accounted for a significant proportion of the heterogeneity.
(TIF)

S5 Fig Effect sizes associated with measures to reduce risk of bias in modelling experiments using other behavioural outcomes. (TIF)

S6 Fig Effect sizes associated with reporting of compliance with animal welfare regulations and a statement of potential conflict of interests in modelling experiments using other behavioural outcomes. (TIF)

S7 Fig Modelling experiments using other behavioural outcomes. (A) Visual inspection of the funnel plot suggests asymmetry. Filled circles represent reported experiments. Solid line represents global effect size, and dashed line represents adjusted global effect size. (B) Trim and fill analysis imputed theoretical missing studies (unfilled circles). Filled circles represent reported experiments. Solid line represents global effect size, and dashed line represents adjusted global effect size. (C) Egger’s regression was not consistent with small study effects.
(TIF)

S8 Fig Impact of study design in intervention experiments using other behavioural outcomes. The size of the squares represents the number of nested comparisons that contribute to that data point, and the value *N* represents the number of animals that contribute to that data point. (A) Type of outcome measure accounted for a significant proportion of the heterogeneity. (B) Strain accounted for a significant proportion of the heterogeneity.
(TIF)

S9 Fig In intervention experiments using other behavioural outcomes, type of intervention accounted for a significant proportion of the heterogeneity. The size of the squares represents the number of nested comparisons that contribute to that data point, and the value *N* represents the number of animals that contribute to that data point.
(TIF)

S10 Fig Effect sizes associated with measures to reduce risk of bias in intervention experiments using other behavioural outcomes. (TIF)

S11 Fig Effect sizes associated with reporting of compliance with animal welfare regulations and a statement of potential conflict of interests in intervention experiments using other behavioural outcomes. (TIF)

S1 File Terms used in each database for systematic search. (DOCX)

## Abbreviations

AITC: TRPA1 agonist allyl isothiocyanate
CIPN: chemotherapy-induced peripheral neuropathy
EDA: Experimental Design Assistant
GRADE: Grading of Recommendations, Assessment, Development and Evaluations
NC3Rs: National Centre for the Replacement, Refinement and Reduction of Animals in Research
NNT: Number Needed to Treat
OSF: Open Science Framework
SD: standard deviation
SE: standard error
SEM: standard error of the mean
SMD: standardised mean difference
