# Supplementary material for: Animal models of chemotherapy-induced peripheral neuropathy: A machine-assisted systematic review and meta-analysis
Source: PLoS Biol. 2019 May 20;17(5):e3000243. doi: 10.1371/journal.pbio.3000243 (PMC6544332; doi:10.1371/journal.pbio.3000243)
Supplement: S1 Table — The median habituation time was 7 days (7–7 IQR). The median number of animals per cage was 4 (2.5–4.5). Reporting of mixed housing with shams was always ‘Not mixed’. Room temperature 22 °C (22 °C–23 °C IQR). Humidity 55 (53.75–55 IQR). (DOCX) [file pbio.3000243.s001.docx]

| Reporting | Diet | Bedding | Cage rack ventilation | Enrichment | Habituation time (days) | Cage cleaning frequency | Number of animals per cage | Housing mixed with sham animals | No. of hours in light cycle | Room temperature | Humidity | Food availability |
| --- | --- | --- | --- | --- | --- | --- | --- | --- | --- | --- | --- | --- |
| No. of publications/337 | 94 | 33 | 4 | 5 | 90 | 3 | 149 | 15 | 279 | 155 | 30 | 289 |
| % | 28 | 10 | 1 | 1 | 26 | <1 | 44 | 4 | 81 | 46 | 9 | 86 |
