# Supplementary figures and images for: Animal models of chemotherapy-induced peripheral neuropathy: A machine-assisted systematic review and meta-analysis
Source: PLoS Biol. 2019 May 20;17(5):e3000243. doi: 10.1371/journal.pbio.3000243 (PMC6544332; doi:10.1371/journal.pbio.3000243)

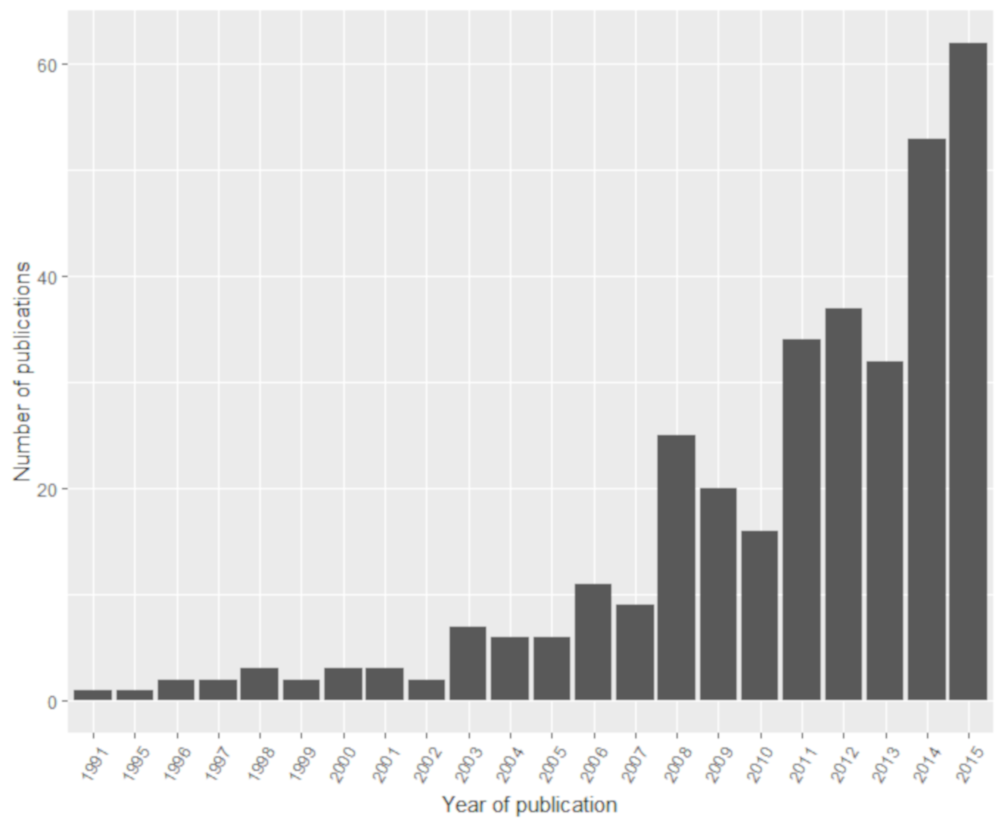

Supplement: S1 Fig — (TIF) [file pbio.3000243.s002.tif]

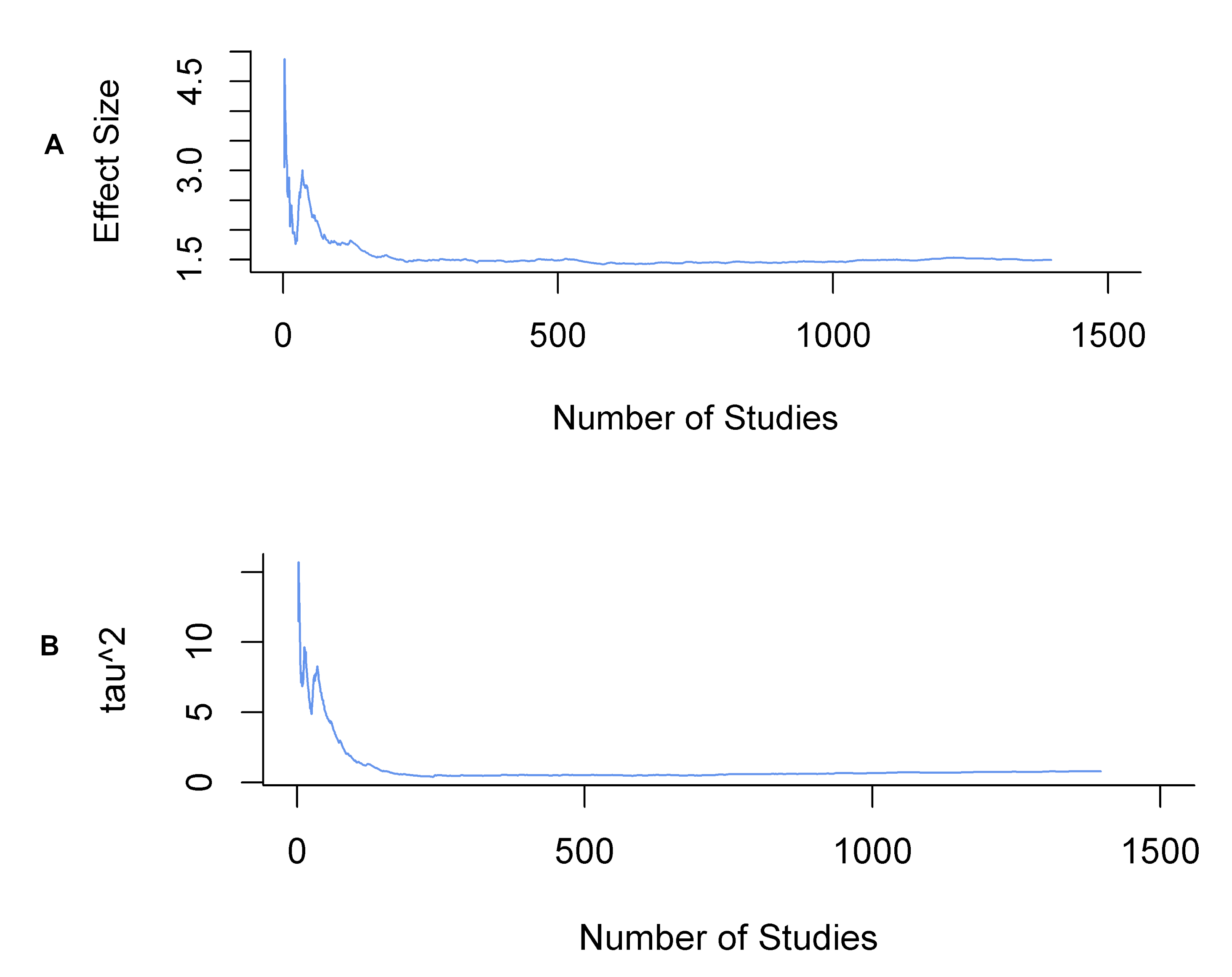

Supplement: S2 Fig — (TIF) [file pbio.3000243.s003.tif]

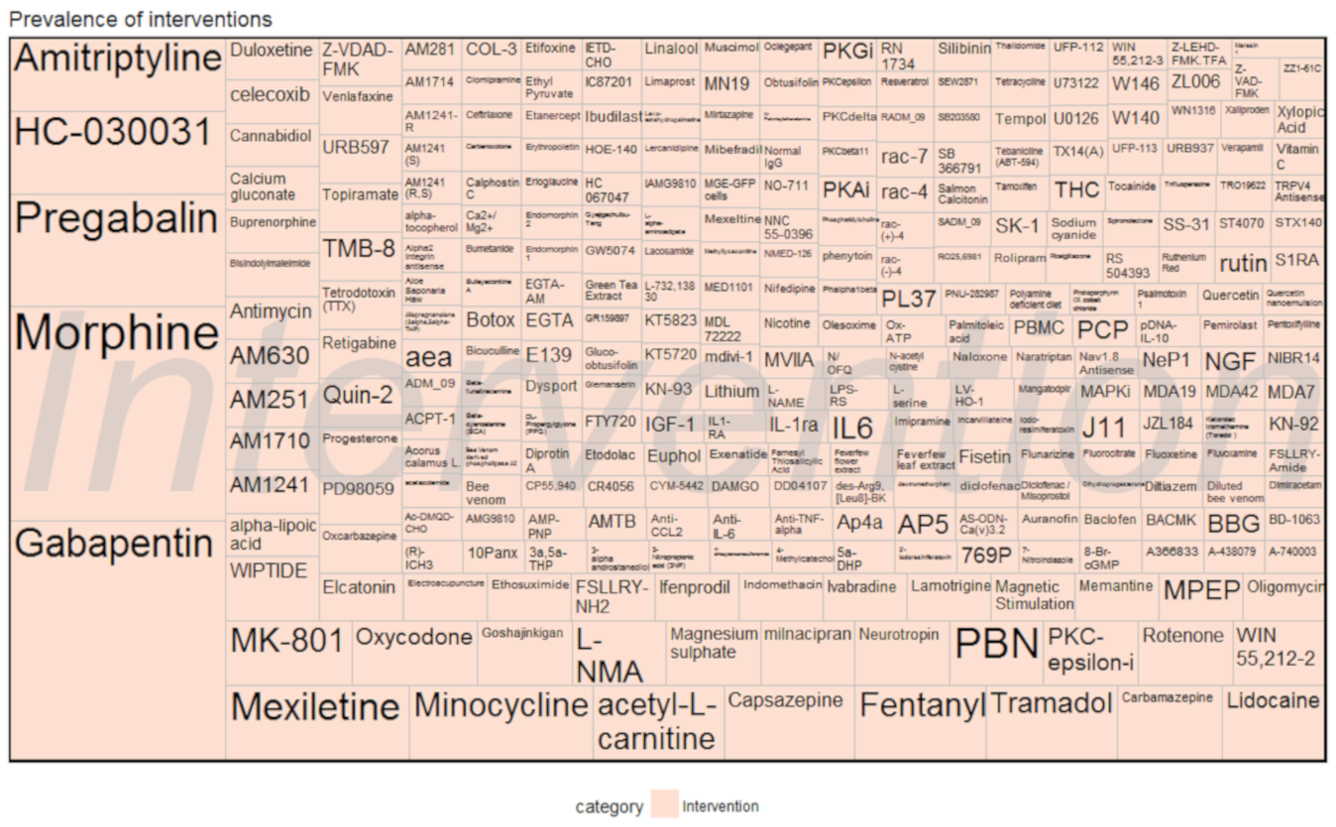

Supplement: S3 Fig — A total of 306 different interventions reported. (TIF) [file pbio.3000243.s004.tif]

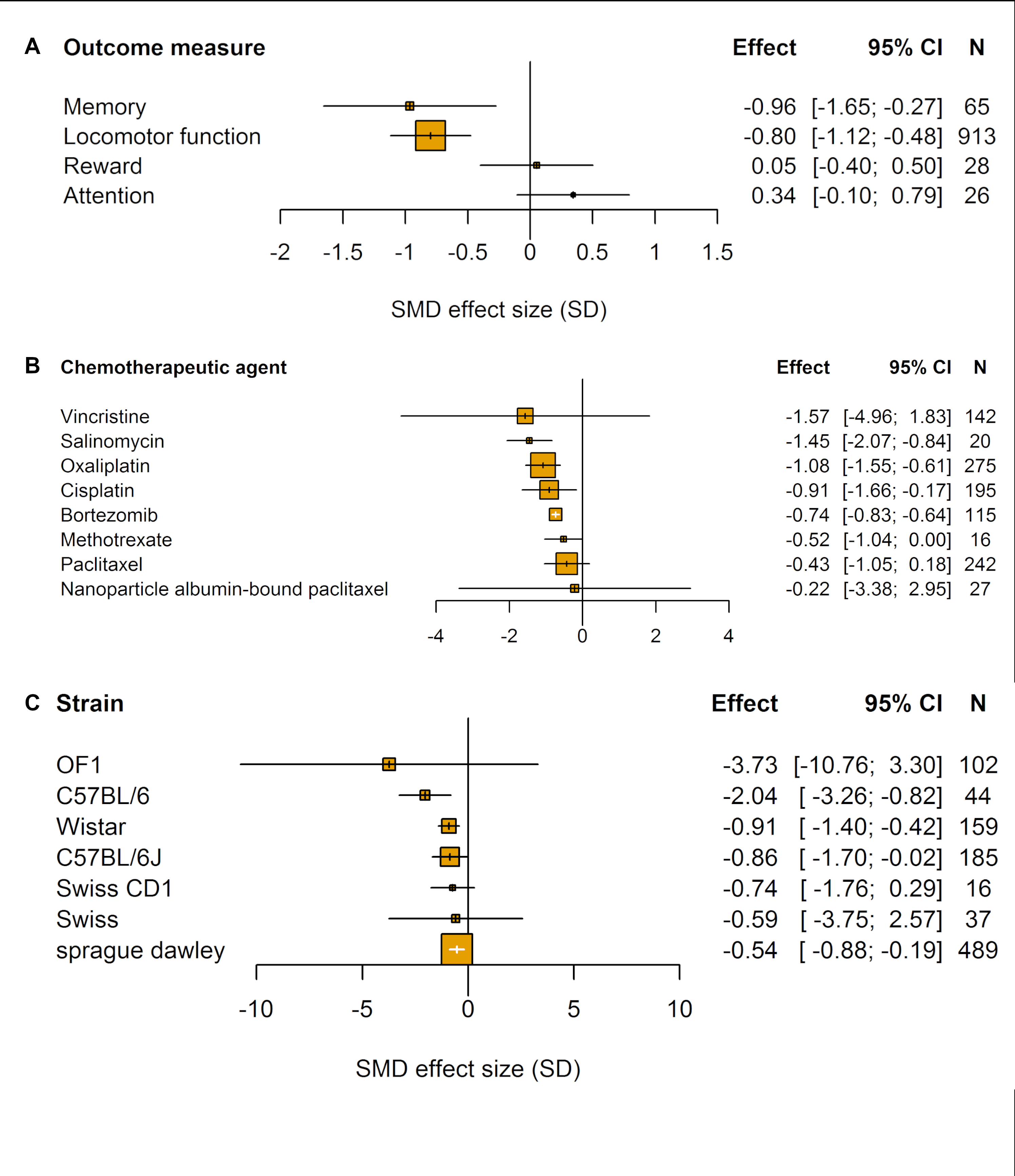

Supplement: S4 Fig — The size of the squares represents the number of nested comparisons that contribute to that data point, and the value N represents the number of animals that contribute to that data point. (A) Outcome measure accounted for a significant proportion of the heterogeneity. (B) Chemotherapeutic agent accounted for a significant proportion of the heterogeneity. (C) Strain accounted for a significant proportion of the heterogeneity. (TIF) [file pbio.3000243.s005.tif]

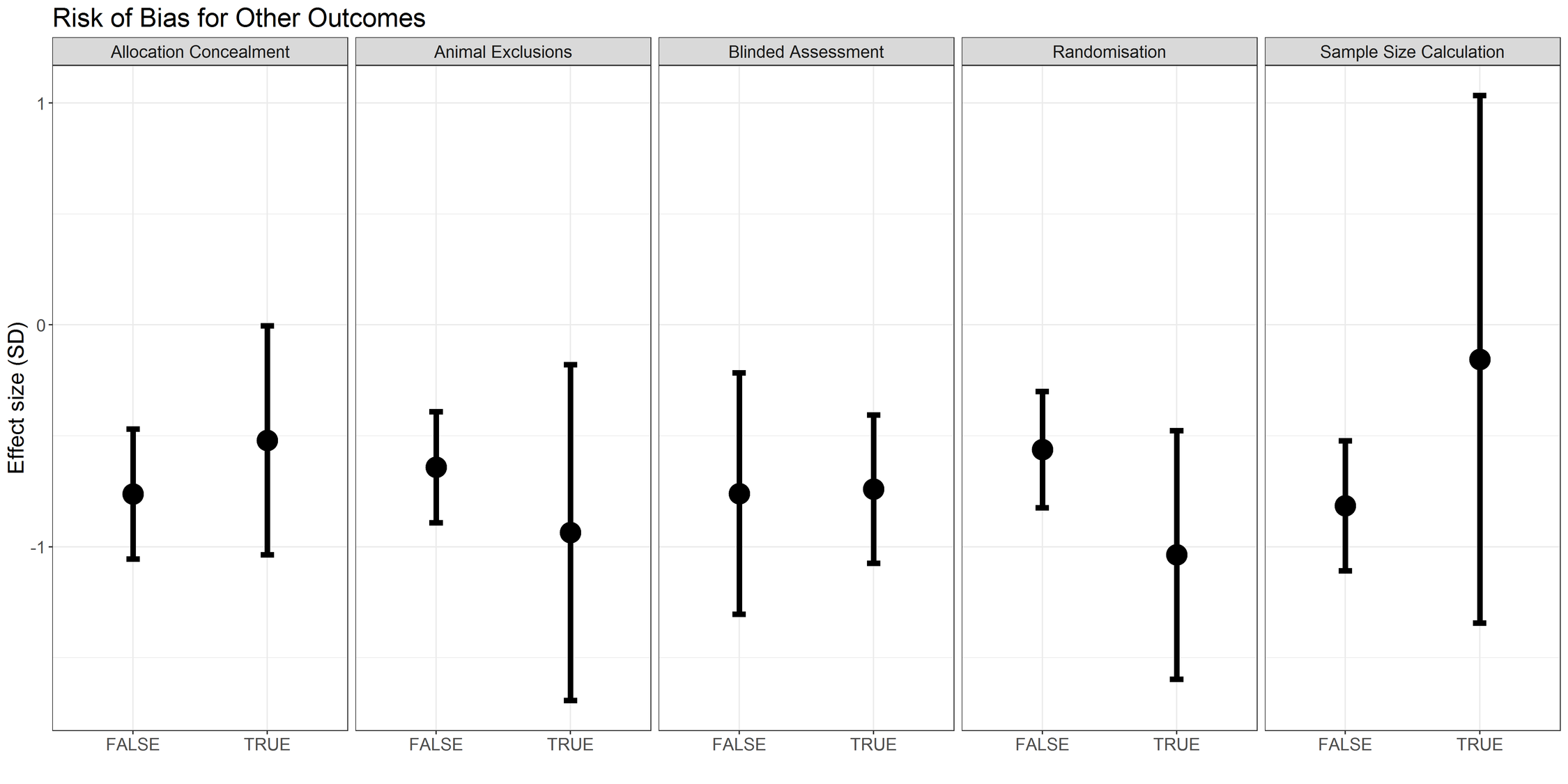

Supplement: S5 Fig — (TIF) [file pbio.3000243.s006.tif]

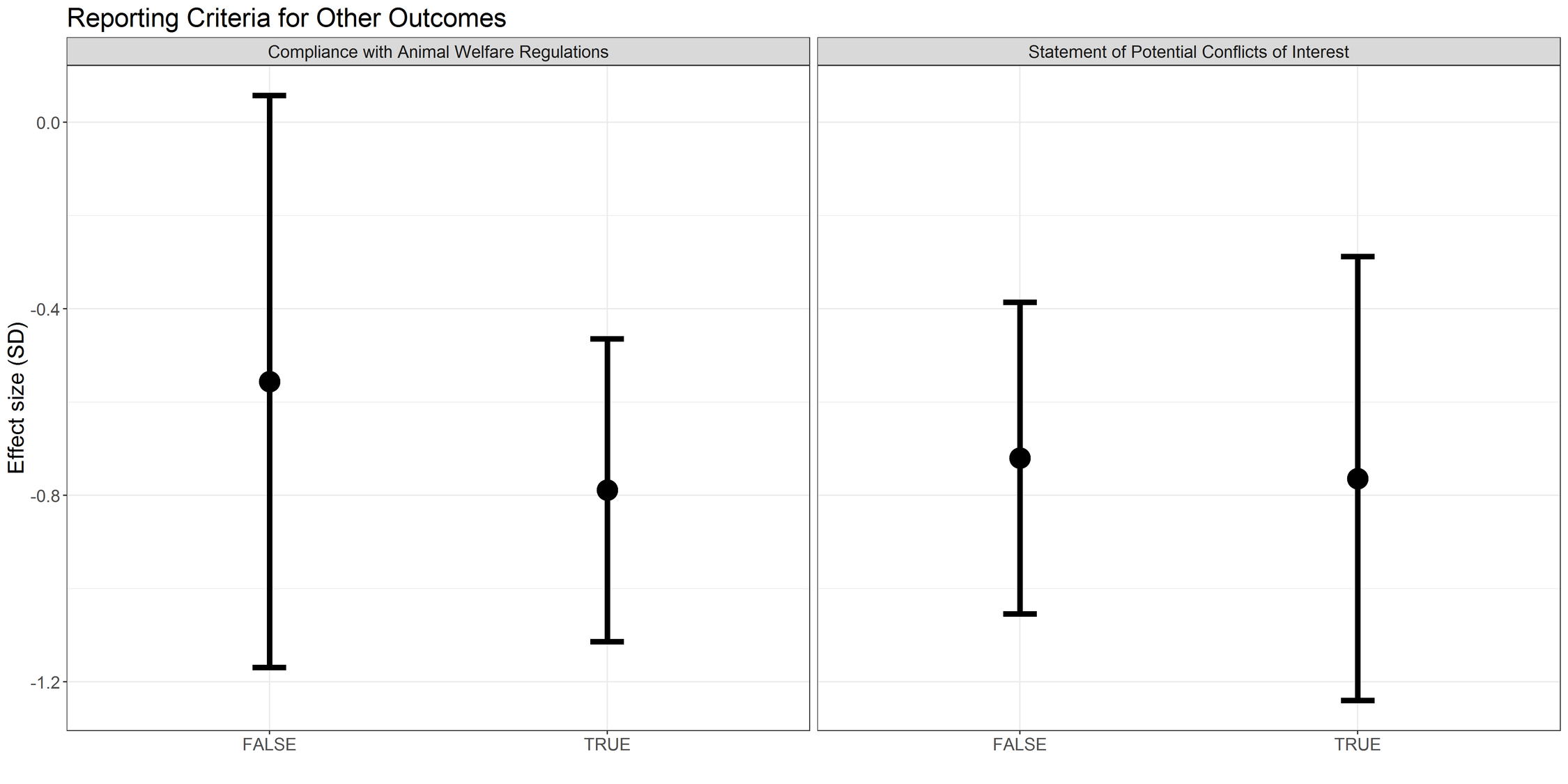

Supplement: S6 Fig — (TIF) [file pbio.3000243.s007.tif]

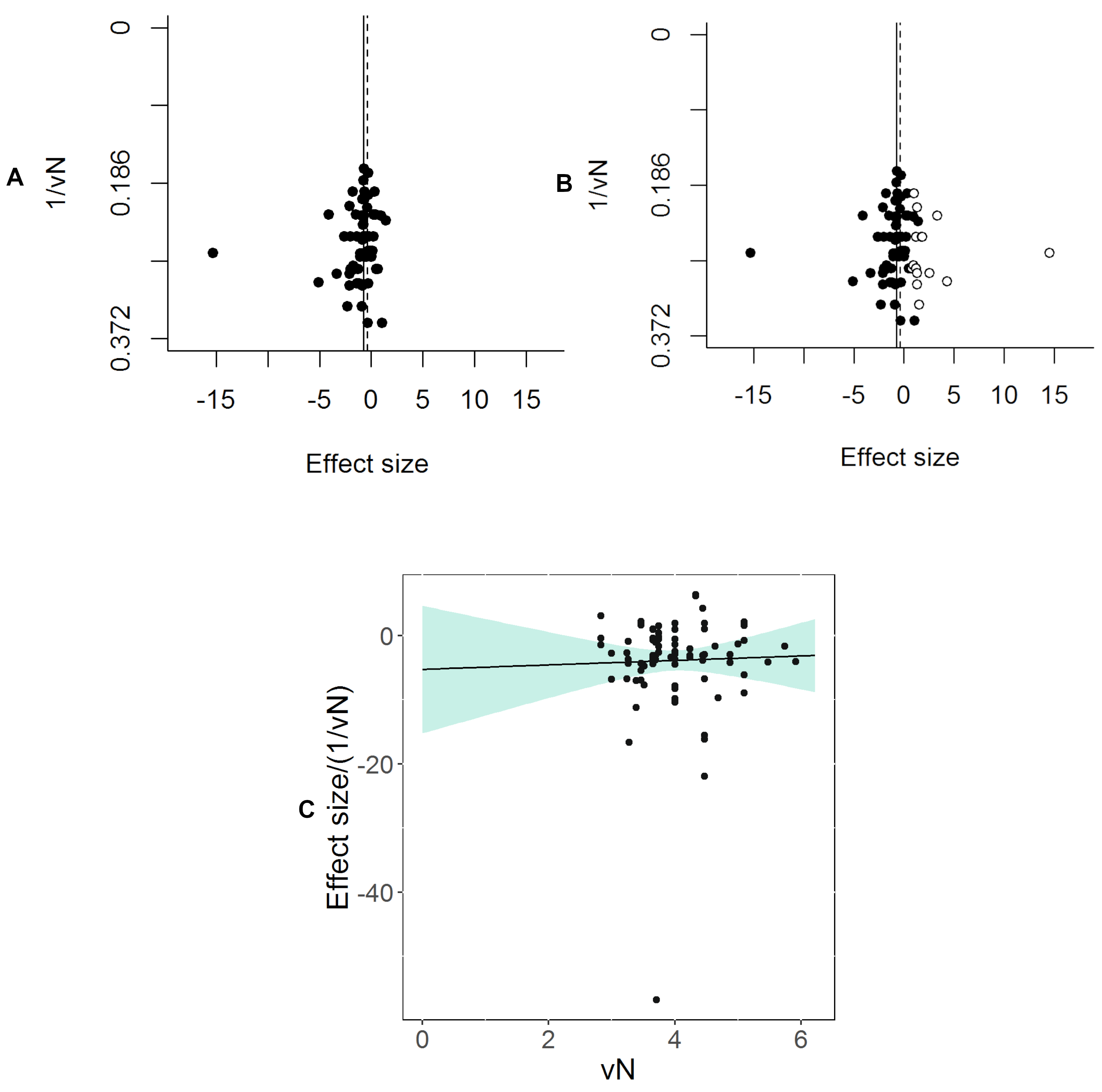

Supplement: S7 Fig — (A) Visual inspection of the funnel plot suggests asymmetry. Filled circles represent reported experiments. Solid line represents global effect size, and dashed line represents adjusted global effect size. (B) Trim and fill analysis imputed theoretical missing studies (unfilled circles). Filled circles represent reported experiments. Solid line represents global effect size, and dashed line represents adjusted global effect size. (C) Egger’s regression was not consistent with small study effects. (TIF) [file pbio.3000243.s008.tif]

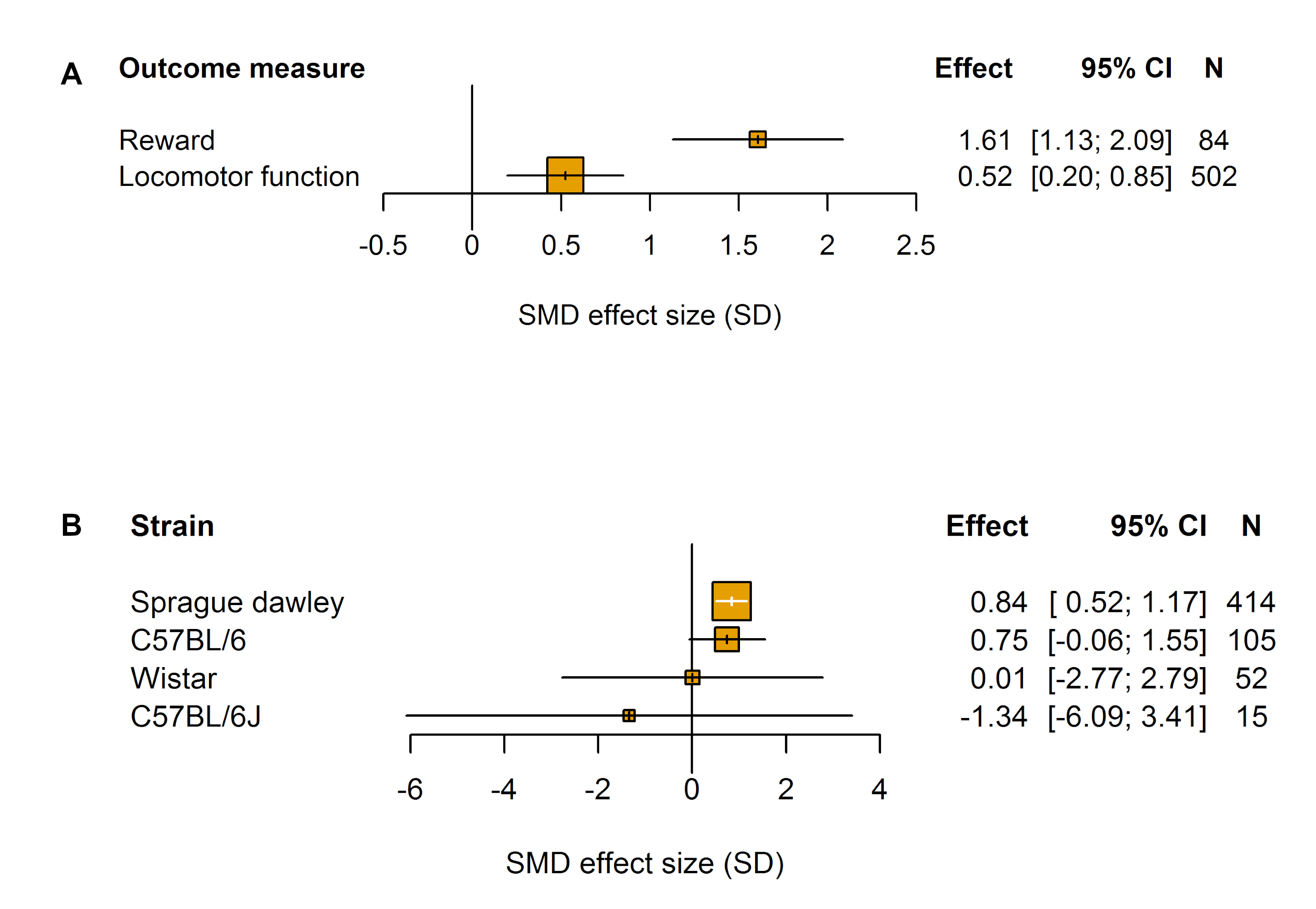

Supplement: S8 Fig — The size of the squares represents the number of nested comparisons that contribute to that data point, and the value N represents the number of animals that contribute to that data point. (A) Type of outcome measure accounted for a significant proportion of the heterogeneity. (B) Strain accounted for a significant proportion of the heterogeneity. (TIF) [file pbio.3000243.s009.tif]

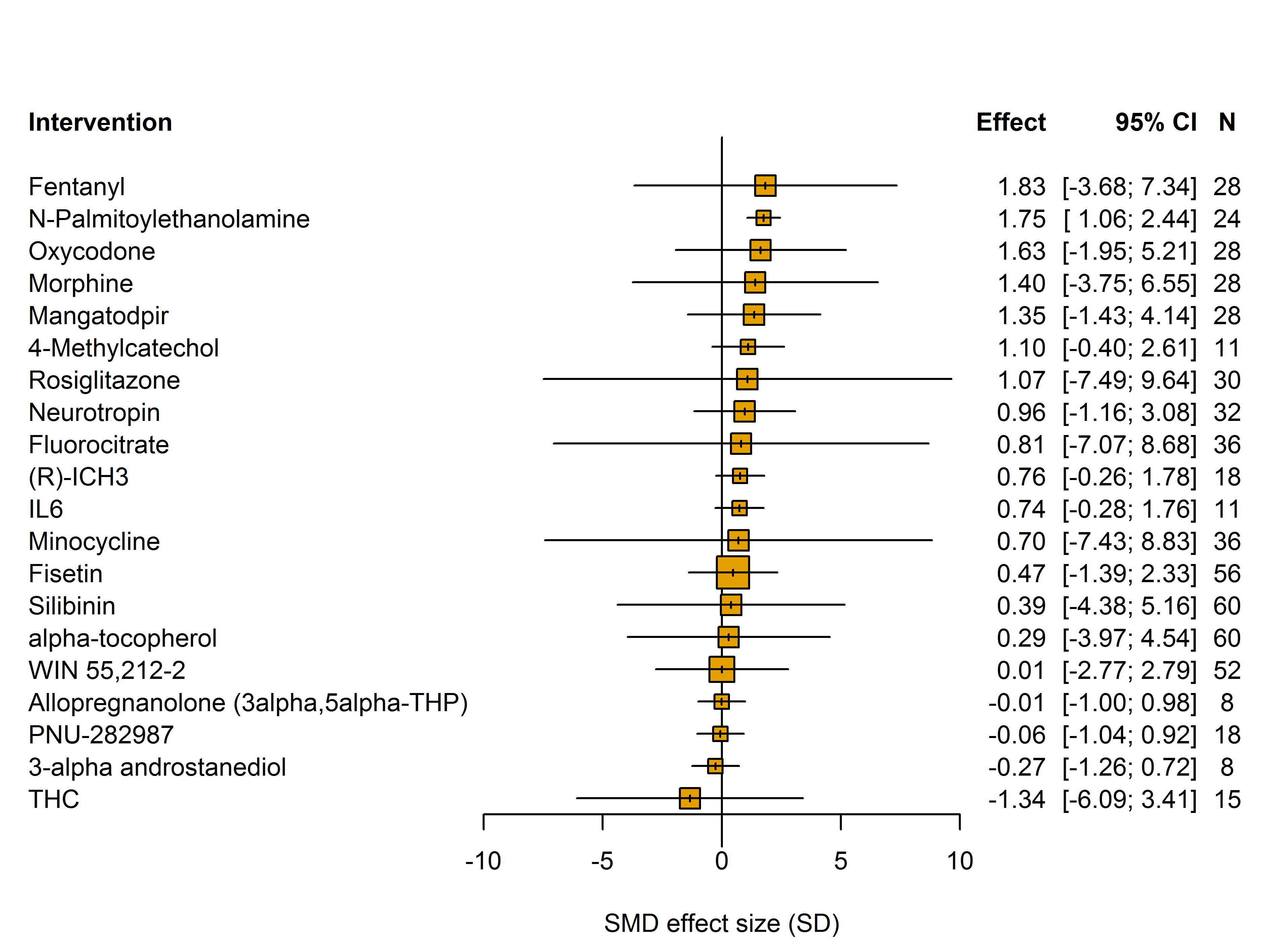

Supplement: S9 Fig — The size of the squares represents the number of nested comparisons that contribute to that data point, and the value N represents the number of animals that contribute to that data point. (TIF) [file pbio.3000243.s010.tif]

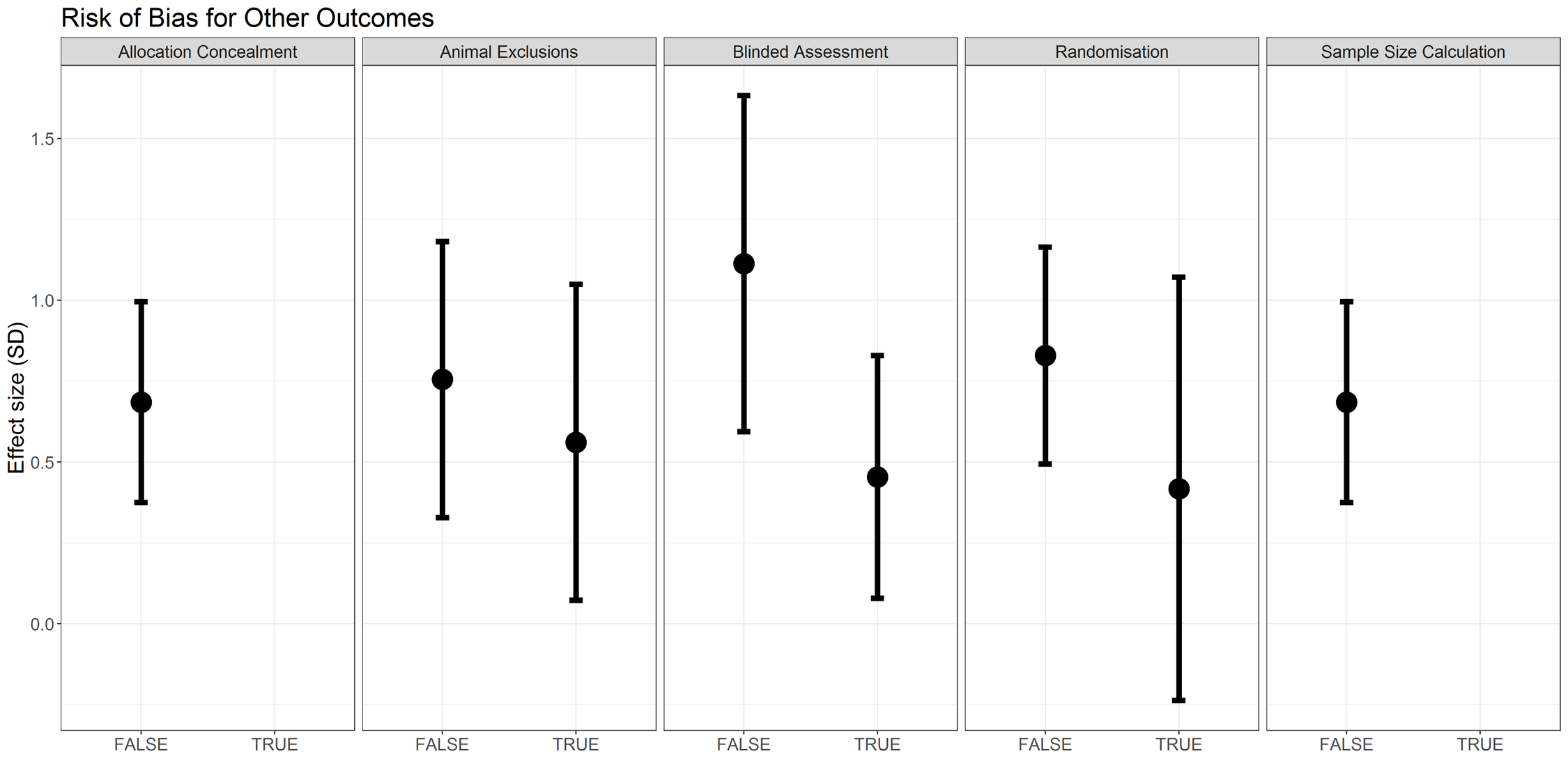

Supplement: S10 Fig — (TIF) [file pbio.3000243.s011.tif]

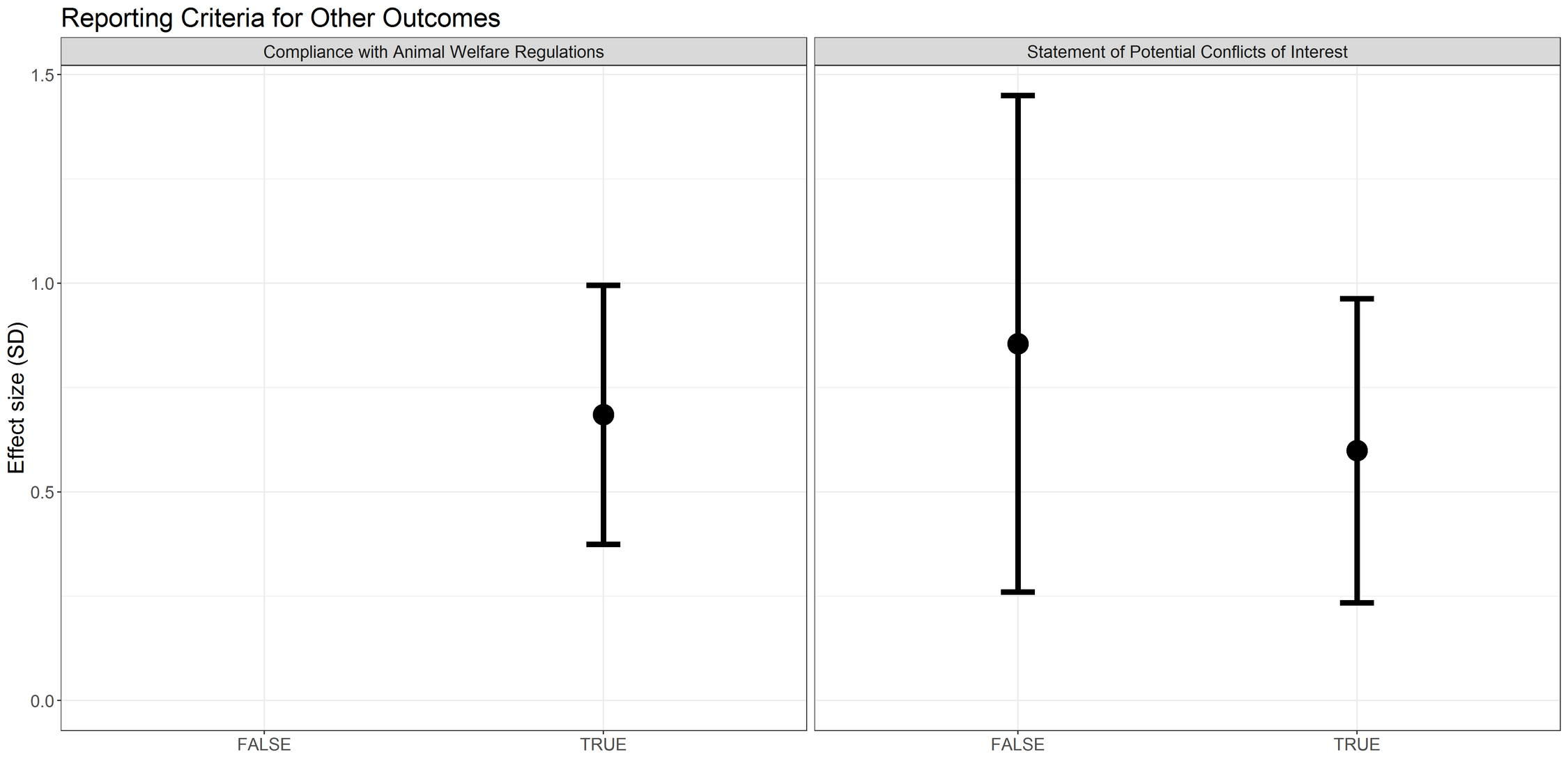

Supplement: S11 Fig — (TIF) [file pbio.3000243.s012.tif]
