# Supplementary material for: Animal models of chemotherapy-induced peripheral neuropathy: A machine-assisted systematic review and meta-analysis
Source: PLoS Biol. 2019 May 20;17(5):e3000243. doi: 10.1371/journal.pbio.3000243 (PMC6544332; doi:10.1371/journal.pbio.3000243)
Supplement: S1 File — (DOCX) [file pbio.3000243.s013.docx]

Pubmed

1. (“neuropathic pain” OR neuropathic OR allodynia OR neuralgic)
2. ("chronic constriction injury" OR "chronic constrictive injury") AND (pain OR analgesia OR analgesic OR allodynia OR neuralgia)
3. (nerve ligation) AND (pain OR analgesia OR analgesic OR allodynia OR neuralgia)
4. (nerve injury) AND (pain OR analgesia OR analgesic OR allodynia OR neuralgia)
5. (nerve compression) AND (pain OR analgesia OR analgesic OR allodynia OR neuralgia)
6. (contusion injury OR hemisection OR dorsal column injury OR corticospinal tract injury OR complete transection OR spinal cord injury) AND (pain OR analgesia OR analgesic OR allodynia OR neuralgia)
7. Search ("nerve transection" OR "transection of nerve") AND (pain OR analgesia OR analgesic OR allodynia OR neuralgia)
8. (chemotherapy[tiab] OR paclitaxel OR oxaliplatin OR taxol) AND (pain OR analgesia OR analgesic OR allodynia OR neuralgia)
9. (streptozotocin OR diabetes [tiab] OR diabetic) AND (pain OR analgesia OR analgesic OR allodynia OR neuralgia)
10. (d4T OR antiretroviral OR anti-retroviral) AND (pain OR analgesia OR analgesic OR allodynia OR neuralgia)
11. (HIV[tiab] OR human immunodeficiency virus[tiab]) AND (pain OR analgesia OR analgesic OR allodynia OR neuralgia)
12. (shingles[tiab] OR postherpetic[tiab] OR herpes zoster[tiab] OR varicella zoster[tiab] OR herpetic[tiab] OR herpes simplex virus[tiab]) AND (pain OR analgesia OR analgesic OR allodynia OR neuralgia)
13. (bone cancer[MeSH] OR bone cancer[tiab] OR cancer of the bone[tiab] OR osteosarcoma[tiab]) AND (pain OR analgesia OR analgesic OR allodynia OR neuralgia)
14. (neuropathy) AND (pain OR analgesia OR analgesic OR allodynia OR neuralgia)
15. (#1 OR #2 OR #3 OR #4 OR #5 OR #6 OR #7 OR #8 OR #9 OR #10 OR #11 OR #12 OR #13 OR #14)
16. #15 AND Animal Study Search Filter [2]

Web of Knowledge (Web of Science, BIOSIS Previews and Biosis Citation Index)

1. TS= ("neuropathic pain" OR neuropathic OR allodynia OR neuralgic)
2. 2. TS= (("chronic constriction injury" OR "chronic constrictive injury") AND (pain OR analgesia OR analgesic OR allodynia OR neuralgia))
3. TS= (("nerve ligation") AND (pain OR analgesia OR analgesic OR allodynia OR neuralgia))
4. TS= (("nerve injury") AND (pain OR analgesia OR analgesic OR allodynia OR neuralgia))
5. TS= (("nerve compression") AND (pain OR analgesia OR analgesic OR allodynia OR neuralgia))
6. TS= (("contusion injury" OR hemisection OR “dorsal column injury” OR “corticospinal tract injury” OR “complete transection” OR “spinal cord injury”) AND (pain OR analgesia OR analgesic OR allodynia OR neuralgia))
7. TS= (("nerve transection" OR "transection of nerve") AND (pain OR analgesia OR analgesic OR allodynia OR neuralgia))
8. TS= ((chemotherapy OR paclitaxel OR oxaliplatin) AND (pain OR analgesia OR analgesic OR allodynia OR neuralgia))
9. TS= ((streptozotocin OR diabetes OR diabetic) AND (pain OR analgesia OR analgesic OR allodynia OR neuralgia))
10. TS= ((d4T OR antiretroviral OR anti-retroviral) AND (pain OR analgesia OR analgesic OR allodynia OR neuralgia))
11. TS= ((HIV OR "human immunodeficiency virus") AND (pain OR analgesia OR analgesic OR allodynia OR neuralgia))
12. TS= ((shingles OR postherpetic OR "herpes zoster" OR "varicella zoster" OR herpetic OR "herpes simplex virus") AND (pain OR analgesia OR analgesic OR allodynia OR neuralgia))
13. TS= (("bone cancer" OR "cancer of the bone" OR CIBP OR "cancer-induced bone pain" OR osteocarcoma) AND (pain OR analgesia OR analgesic OR allodynia OR neuralgia))
14. TS= ((neuropathy) AND (pain OR analgesia OR analgesic OR allodynia OR neuralgia))
15. (#1 OR #2 OR #3 OR #4 OR #5 OR #6 OR #7 OR #8 OR #9 OR #10 OR #11 OR #12 OR #13 OR #14)
16. #15 AND Adapted Animal Study Search Filter [2]
17. Taxa Notes=(Nonhuman Mammals OR Nonhuman Primates OR Nonhuman Vertebrates)
18. #15 AND #17

Web of Science

#16

Refined by: [excluding] Document Types=( EDITORIAL MATERIAL OR BOOK CHAPTER OR BIOGRAPHICAL ITEM OR REVIEW OR NOTE OR LETTER OR NEWS ITEM )

BIOSIS Previews

#18

Biosis Citation Index

#18

Refined by: [excluding] Document Types=( BOOK OR LETTER OR BOOK CHAPTER OR PATENT )

Embase

1. (neuropathic pain/ OR neuropathic OR allodynia OR neuralgic)
2. ((chronic constriction injury/ OR chronic constrictive injury/) AND (pain OR analgesia OR analgesic OR allodynia OR neuralgia))
3. ((nerve ligation/) AND (pain OR analgesia OR analgesic OR allodynia OR neuralgia))
4. ((nerve injury/) AND (pain OR analgesia OR analgesic OR allodynia OR neuralgia))
5. ((nerve compression/) AND (pain OR analgesia OR analgesic OR allodynia OR neuralgia))
6. ((contusion injury/ OR hemisection OR dorsal column injury/ OR corticospinal tract injury/ OR complete transection/ OR spinal cord injury/) AND (pain OR analgesia OR analgesic OR allodynia OR neuralgia))
7. (("nerve transection" OR "transection of nerve") AND (pain OR analgesia OR analgesic OR allodynia OR neuralgia))
8. ((chemotherapy OR paclitaxel OR oxaliplatin) AND (pain OR analgesia OR analgesic OR allodynia OR neuralgia))
9. ((streptozotocin OR diabetes OR diabetic) AND (pain OR analgesia OR analgesic OR allodynia OR neuralgia))
10. ((d4T OR antiretroviral OR anti-retroviral) AND (pain OR analgesia OR analgesic OR allodynia OR neuralgia))
11. ((HIV OR human immunodeficiency virus/) AND (pain OR analgesia OR analgesic OR allodynia OR neuralgia))
12. ((shingles OR postherpetic OR herpes zoster/ OR varicella zoster/ OR herpetic OR herpes simplex virus/) AND (pain OR analgesia OR analgesic OR allodynia OR neuralgia))
13. ((bone cancer/ OR cancer of the bone/ OR CIBP OR cancer-induced bone pain/ OR osteocarcoma) AND (pain OR analgesia OR analgesic OR allodynia OR neuralgia))
14. ((neuropathy) AND (pain OR analgesia OR analgesic OR allodynia OR neuralgia))
15. (#1 OR #2 OR #3 OR #4 OR #5 OR #6 OR #7 OR #8 OR #9 OR #10 OR #11 OR #12 OR #13 OR #14)
16. #15 AND Animal Study Search Filter [1]
17. #16 Limit to embase.
18. #17 Limit to (article or conference abstract or conference paper or erratum or journal or conference proceeding or report)

[1] de Vries RBM, Hooijmans CR, Tillema A, Leenaars M, Ritskes-Hoitinga M. A search filter for increasing the retrieval of animal studies in Embase. Laboratory animals 2011;45(4):268-270.

[2] Hooijmans CR, Tillema A, Leenaars M, Ritskes-Hoitinga M. Enhancing search efficiency by means of a search filter for finding all studies on animal experimentation in PubMed. Laboratory animals 2010;44(3):170-175.
